# *Serpine1* mRNA confers mesenchymal characteristics to the cell and promotes CD8+ T cells exclusion from colon adenocarcinomas

**DOI:** 10.1038/s41420-024-01886-8

**Published:** 2024-03-06

**Authors:** Salvador Polo-Generelo, Cristina Rodríguez-Mateo, Belén Torres, José Pintor-Tortolero, José A. Guerrero-Martínez, Julian König, Jesús Vázquez, Elena Bonzón-Kulichenco, Javier Padillo-Ruiz, Fernando de la Portilla, José C. Reyes, José A. Pintor-Toro

**Affiliations:** 1 Department of Cell Signaling, Centro Andaluz de Biología Molecular y Medicina Regenerativa (CABIMER-CSIC), 41092 Sevilla, Spain; 2 Colorectal Surgery Unit, Department of General and Digestive Surgery, Virgen del Rocío University Hospital, IBIS, CSIC, University of Sevilla, Sevilla, Spain; 3 Institute of Molecular Biology (IMB), Ackermannweg 4, 55128 Mainz, Germany; 4 Cardiovascular Proteomics, Centro Nacional de Investigaciones Cardiovasculares (CNIC), 28029 Madrid, Spain; 5 Facultad de Ciencias Ambientales y Bioquímica, Universidad de Castilla-La Mancha, Toledo, Spain; 6 Hepatobiliary Surgery Unit, Department of General and Digestive Surgery, Virgen del Rocío University Hospital, IBIS, CSIC, University of Sevilla, Sevilla, Spain

**Keywords:** Long non-coding RNAs, Colon cancer, Immunosurveillance, Immune evasion

## Abstract

Serine protease inhibitor clade E member 1 (SERPINE1) inhibits extracellular matrix proteolysis and cell detachment. However, SERPINE1 expression also promotes tumor progression and plays a crucial role in metastasis. Here, we solve this apparent paradox and report that *Serpine1* mRNA per se, independent of its protein-coding function, confers mesenchymal properties to the cell, promoting migration, invasiveness, and resistance to anoikis and increasing glycolytic activity by sequestering miRNAs. Expression of *Serpine1* mRNA upregulates the expression of the TRA2B splicing factor without affecting its mRNA levels. Through transcriptional profiling, we found that *Serpine1* mRNA expression downregulates through TRA2B the expression of genes involved in the immune response. Analysis of human colon tumor samples showed an inverse correlation between *SERPINE1* mRNA expression and CD8+ T cell infiltration, unveiling the potential value of *SERPINE1* mRNA as a promising therapeutic target for colon tumors.

## Introduction

During the Epithelial-Mesenchymal Transition (EMT) process, cells undergo reversible phenotypic changes, losing their epithelial characteristics and acquiring attributes of mesenchymal cells. In this process, apical-basolateral polarity is lost, cell-cell junctions are disrupted, the actin cytoskeleton is extensively reorganized, and the cells acquire mesenchymal characteristics, including a migratory and invasive phenotype [1, 2]. EMT is essential for embryonic development and wound healing and has been associated with pathological processes such as organ fibrosis and cancer progression [3].

Transcriptional regulation is widely recognized as the primary level of regulation of EMT. However, it is increasingly evident that EMT is a complex biological process orchestrated by multiple layers of regulation. Post-transcriptional mechanisms, such as regulation by alternative pre-mRNA splicing and regulation by miRNAs, provide additional layers of complexity to the gene regulation during EMT. Alternative splicing (AS) produces different protein isoforms that orchestrate essential aspects of the EMT process, including cell-cell contacts, polarity, and cytoskeleton organization. These AS events are coordinately regulated by specific alternative splicing factors. While ESRP1 and ESRP2 are fundamental to establishing an epithelial-specific splicing program, RBFOX2 and MBNL1 are essential contributors to the mesenchymal splicing signatures [4, 5]. MicroRNAs (miRNAs) are small non-coding RNAs that post-transcriptionally regulate gene expression [6]. RNA polymerase II initially transcribes them as longer transcripts known as pri-miRNAs. They are processed in the nucleus by the RNase III nuclease Drosha into ∼70 nt pre-miRNAs and processed in the cytoplasm by Dicer nuclease into miRNA duplexes of 18-24 nt. One of the strands of the mature miRNA called the guide strand, associates with the Argonaute2 (AGO2) protein and guides the RNA-induced silencing complex (RISC) to the target transcript, inducing its translational repression or mRNA degradation [7]. Crosslinking and immunoprecipitation (CLIP) methods [8] have identified AGO binding sites at a transcriptome-wide scale, generating context-dependent AGO binding maps. As a result of recent technological advances, parameters that determine miRNA‐mediated gene regulation and readouts (such as mRNA abundance) can now be measured with high resolution. These have provided the opportunity to study quantitatively, using computational models, how the interplay of regulators and targets gives rise to the observed gene expression patterns. In this work, we have studied the EMT post-transcriptional regulation by analyzing and quantifying the RNAs enriched in the RISC complex at the early stages of the EMT process.

Here, we show that the most enriched RNA in the RISC complex during the EMT process is the *Serpine1* mRNA. Paradoxically, this mRNA is strongly transcribed from the start of the EMT process [9]. SERPINE1 inhibits fibrinolysis by blocking tissue plasminogen activator (tPA) and urokinase (uPA), which deactivate plasminogen-induced extracellular matrix degradation [10]. In addition, SERPINE1 binds to vitronectin, stabilizing it, and interferes with the binding of integrins to vitronectin, affecting cell migration [11–13]. Previous research has mainly concentrated on the function of SERPINE1 in thrombogenesis. Recent research has shown that a high SERPINE1 concentration is a bad prognostic factor in lung, neuroblastoma, breast, gastric, and colorectal cancers [14–19]. Interestingly, high SERPINE1 expression has been shown to enhance cell migration and apoptosis resistance in head and neck carcinoma patients, which may explain the association between high SERPINE1 expression and poor outcomes in these patients [20]. On the other hand, down-regulation of SERPINE1 in nasopharyngeal carcinoma has been associated with reduced metastasis [21]. Furthermore, cocultured metastatic melanoma and triple-negative breast cancer cells with species-homologous platelets displayed more robust capabilities for cell migration, invasion, and colony formation in vitro and also tumor growth and metastasis in vivo. Transcriptomic analysis showed that co-cultured cancer cells had high levels of *SERPINE1*. Knockdown of *Serpine1* reversed cancer cells’ survival and metastatic abilities [22]. SERPINE1 expression would be expected to have a protective effect against tumor dissemination by inhibiting extracellular matrix proteolysis and cell detachment. However, most of the studies conducted to date reveal that SERPINE1 has a paradoxical effect on cancer, promoting tumor progression, increasing the risk of metastasis, and supporting a multifunctional role for Serpine1.

Here, we present data that could explain this apparent *Serpine1* paradox. We demonstrate in a mouse mammary gland cell line (NMuMG) that *Serpine1* mRNA acts as a non-coding RNA, sequestering miRNAs, impeding their repressive activity, and causing phenotypic effects hitherto associated with SERPINE1 protein. We also show that the *Serpine1* mRNA downregulates, through the TRA2B splicing factor, the expression of genes primarily associated with the immune response. Furthermore, our study reveals an inverse association between human *SERPINE1* mRNA expression and the presence of CD8^+^ T cells in colon adenocarcinomas, bringing about the inhibition of *SERPINE1* mRNA as a valuable therapeutic strategy to increase T cell infiltration in colon tumors.

## Results

### *Serpine1* mRNA is enriched in the RISC complex during TGF-β-mediated EMT

**Fig. 1:**
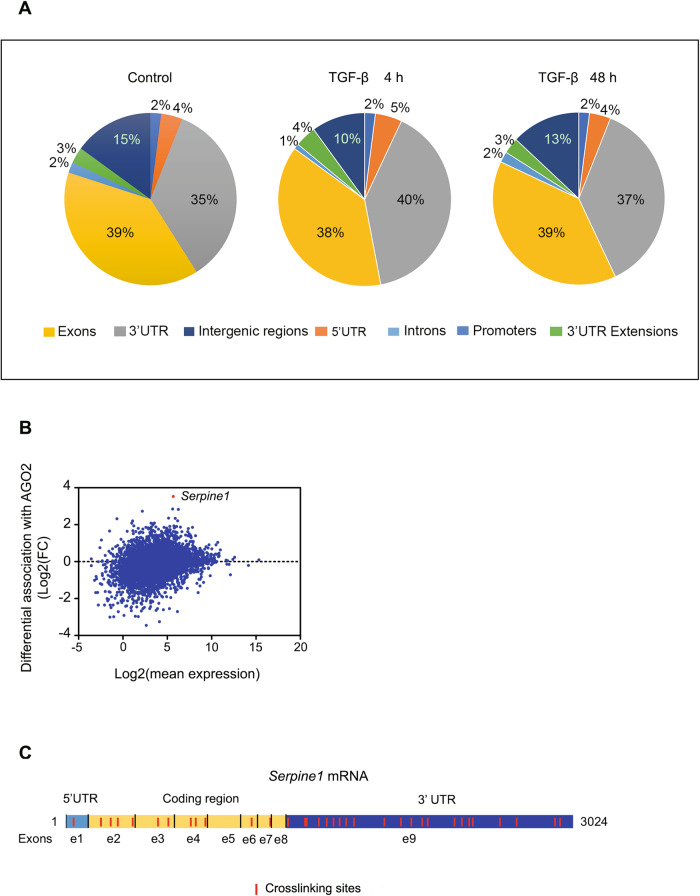
Argonaute iCLIP. **A** Location of AGO2-mRNA tags in the genome of control NMuMG cells and cells treated for 4, and 48 h with TGF-β. **B** Differential association of transcripts to AGO2 after 4 h of TGF-β treatment [Log_2_(Fold Change 4 h of TGF-β versus Control)], respect to transcript mean expression. The *Serpine1* point was highlighted in red. **C** Distribution of the AGO2 34 crosslinking sites along the *Serpine1* mRNA. 3´UTR, 5´UTR, coding region and exons of the *Serpine1* mRNA are indicated.

RNA-induced silencing complex (RISC) is a basic eukaryotic cellular machinery that plays a pivotal role in post-transcriptional gene regulation. AGO2 protein is an essential component of the RISC and directly binds miRNAs. We hypothesized that increased or decreased RNAs early in the RISC complex during EMT could represent putative EMT regulators. We used NMuMG cells as a model system [1] to identify and quantify the RNA changes in the RISC complex during EMT. This non-transformed mouse mammary epithelial cell line performs a robust and reproducible TGF-β-induced transition from an epithelial to a mesenchymal state. Cells were treated with TGF-β for 4 and 48 h, UV-crosslinked, and the RISC complex was coimmunoprecipitated using antibodies against AGO2. RNAs from the RISC complex were isolated in triplicates and subjected to deep sequencing (iCLIP-seq). Given the close correlation between the replicates (Supplementary Fig. S1), the reads of the three experiments were merged. Approximately 3.99 million sequenced RNA tags from three libraries of untreated cells, 4.98 million tags from three libraries of 4 h-TGF-β-treated cells, and 3.89 million tags from three libraries of 48 h-TGF-β-treated cells were processed and mapped to the mouse genome. The results showed that the AGO2 protein binds mainly to exons and 3’ UTRs regions, with the percentage of unions to these regions very similar, indicating that miRNA binding sites in coding sequence (CDS) are widespread and almost as common as in 3’UTRs (Fig. 1A). The crosslinking sites and the corresponding reads of each of the RNAs bound to AGO2 in each condition were identified using the PureCLIP software [2] (Supplementary Tables S1 and S2). We focused on RNAs that are most enriched in the RISC complex after 4 hours of treatment with TGF-β, which could be involved in the post-transcriptional regulation of EMT. *Serpine1* mRNA was the most differentially RISC complex-associated mRNA after 4 hours of treatment with TGF-β (Fig. 1B and Table 1). This mRNA also showed the most significant increase in crosslinking sites, going from 2 sites in untreated cells to 34 in TGF-β-treated cells (Table 2; Fig. 1C, Supplementary Fig. S2, and Supplementary Table S3).

**Table 1:** RISC complex-associated mRNAs in TGF-β-treated cells for 4 h.

Gene	Control cells Reads (RPKM)	TGF-β-treated cells Reads (RPKM)	Enrichment
*Serpine1*	74.74	863.58	11.55
*CTGF*	64.66	328.14	5.07
*Bhlhe40*	41.60	192.83	4.63
*Cdh6*	84.68	307.37	3.62
*Pmepa1*	79.03	273.24	3.45
*Bmf*	49.87	153.48	3.08
*Tgfbr1*	92.17	243.53	2.62

*RPKM* Reads Per Kilobase Million.

**Table 2:** Crosslinking sites of RISC complex-associated mRNAs in control cells and TGF-β-treated cells for 4 h.

Gene	Control cells Crosslinking sites	TGF-β-treated cells Crosslinking sites
*Serpine1*	2	34
*CTGF*	2	21
*Bhlhe40*	0	20
*Cdh6*	3	25
*Pmepa1*	4	20
*Bmf*	0	10
*Tgfbr1*	2	31

### *Serpine1* mRNA confers mesenchymal characteristics

**Fig. 2:**
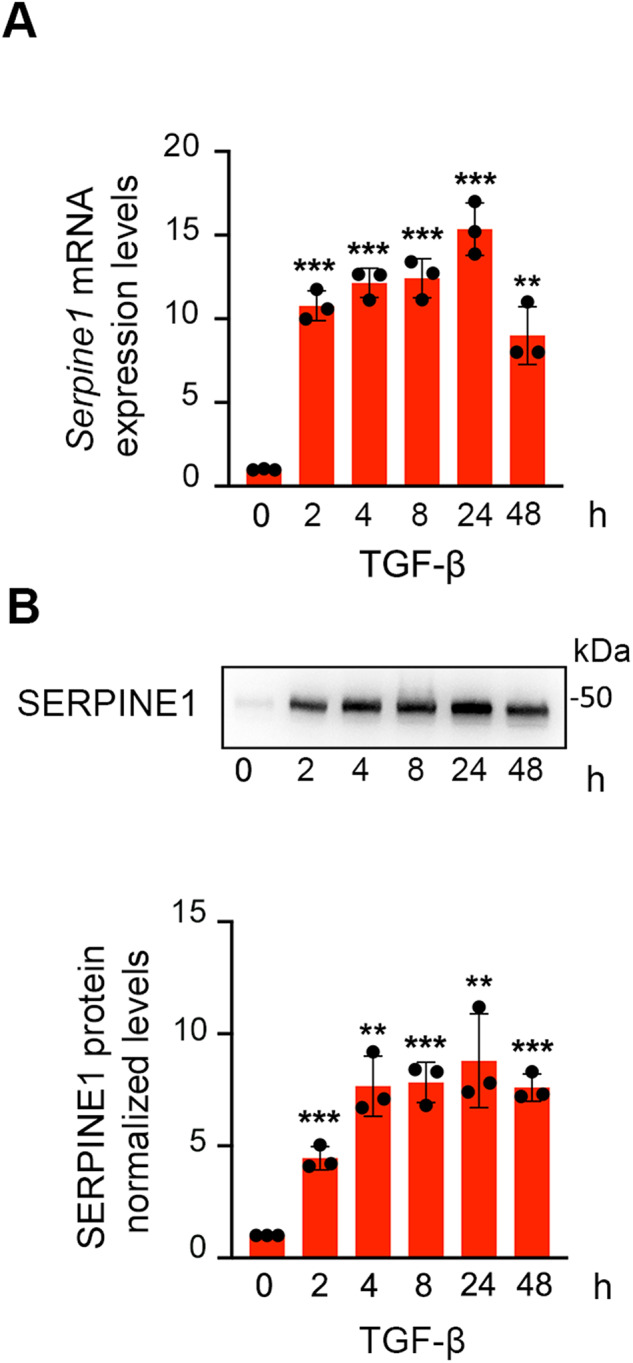
*Serpine1* expression in TGF-β–treated NMuMG cells. Relative levels of mRNA *Serpine1* (**A**) and protein SERPINE1 (**B**) in cells treated with TGF-β at the indicated times. A representative immunoblotting of SERPINE1 is shown. Quantification of *Serpine1* and SERPINE1 in three independent experiments is shown. Error bars represent S.D. ***p < 0.001, **p < 0.01 by two-tailed Student´s *t* test. Protein-loading normalization was performed by measuring total protein directly on the membrane using the criterion stain-free gel imaging system.

**Fig. 3:**
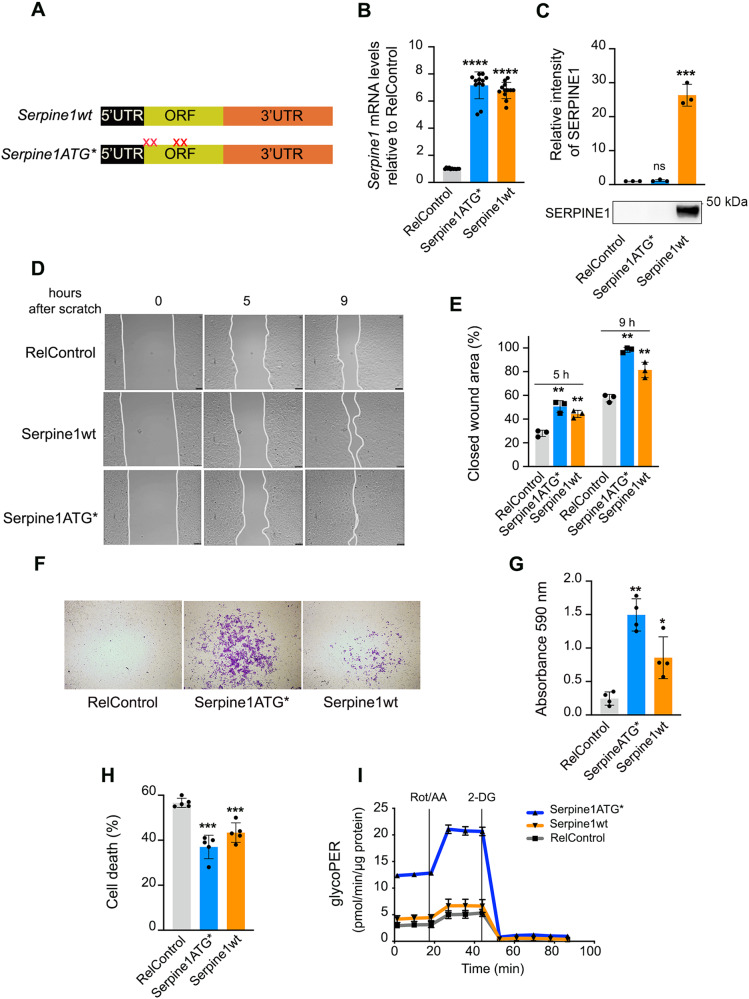
*Serpine1* mRNA upregulation confers mesenchymal characteristics to NMuMG cells. **A** Diagram of the complete Serpine1 cDNA (Serpine1wt) and the cDNA with the start codon and three additional ATG codons mutated (Serpine1ATG*). ORF, open reading frame. Xs represent mutations of the ATG codons in the Serpine1 ORF. **B** Real-time PCR analysis of Serpine1 levels in cells infected at MOI 2 with pHRSIN-DUAL lentivirus control (RelControl), carrying Serpine1wt or Serpine1ATG*. Experiments were repeated fourfold in triplicate samples; error bars represent S.D. ****P < 0.0001 by two-tailed Student´s *t* test. **C** Immunoblotting of SERPINE1 in RelControl, Serpine1ATG* and Serpine1wt cells. Protein-loading normalization was performed by measuring total protein directly on the membrane using the criterion stain-free gel imaging system. Experiments were repeated threefold; error bars represent S.D. ***P < 0.001 by two-tailed Student´s *t* test; ns not significant. **D** The migratory capacity of RelControl, Serpine1ATG*, and Serpine1wt cells was tested in wound-healing assays. Cells were imaged at 10-min intervals for 24 h. The frames of the movie at 0, 5, and 9 h are shown. **E** The frames at 5 and 9 h were used to estimate the percentage of surface covered by the cells. Values represent the average (%) of wound closure. Error bars represent S.D. **P < 0.01 by two-tailed Student’s *t* test. **F** Representative images of invasion of RelControl, Serpine1ATG*, and Serpine1wt cells were analyzed in transwell invasion assays. After 24 h, cells were fixed and stained with crystal violet. Invasive cells were solubilized with 1% SDS at room temperature for 30 min and quantified at O.D. 590 nm. **G** Histograms represent the mean from four independent invasion assays. Error bars represent S.D. **P < 0.01, *P < 0.05 by two-tailed Student’s *t* test. **H** The percentage of sub-G1 cells was determined by flow cytometry. Results are the averages of five independent experiments and were analyzed by one-way ANOVA, followed by the Bonferroni post-test for significance versus control cells. Error bars represent S.D. ***P < 0.001. **I** Proton efflux rate derived from glycolysis (glycoPER) of RelControl, Serpine1ATG*, and Serpine1wt cells. Rot/AA rotenone/antimycin, 2-DG 2-deoxy-D-glucose. GlycoPER is expressed as pmol/min and was corrected using total µg of protein. Data from three independent experiments. Error bars represent S.D.

Transcription of the *Serpine1* gene is strongly and rapidly induced in TGF-β-treated cells. After 4 h of TGF-β treatment, *Serpine1* mRNA levels increased 11-fold, detecting the presence of SERPINE1 protein; however, paradoxically, the amount of *Serpine1* RNA in the RISC complex increased 11.5-fold as well (Fig. 2 and Supplementary Tables S2 and S4) indicating that *Serpine1* mRNA could exert a non-coding biological function, in addition to encoding SERPINE1 protein. We postulated that *Serpine1* mRNA may act as a natural miRNAs sponge, dampening the EMT inhibitor activity of miRNAs. To determine whether *Serpine1* mRNA can operate as a competitive endogenous RNA (ceRNA), stable NMuMG cell lines were generated overexpressing *Serpine1* mRNA (Serpine1wt) and *Serpine1* mRNA mutated at four ATGs (Serpine1ATG*) to prevent its translation, two of them corresponding to the translation start site (TSS), and the other ones located at 800 bp from the TSS (Fig. 3A). Levels of *Serpine1* mRNA in the generated cell lines were similar; however, overexpression of SERPINE1 protein was only found in Serpine1wt cells (Fig. 3B, C). We compared the migratory and invasive abilities in the generated cell lines and observed greater migratory and invasive abilities in Serpine1wt cells compared to the control cells. These abilities were even increased in Serpine1ATG* cells (Fig. 3D–G and Supplementary Movie S1). Comparable proliferative capacities of cells were observed, ruling out that the enhanced migratory capacity and invasiveness were due to differences in the proliferative capacity of these cells (Supplementary Fig. S3). EMT is crucial for epithelial-derived cells to acquire anoikis resistance [3]. The apoptotic cells were quantified in Serpine1ATG* and Serpine1wt cells growing in the presence of methylcellulose and on Poly-HEMA-coated plated to prevent both adherence among them and their adherence to plastic, thereby eliciting anoikis. Serpine1ATG* and Serpine1wt cells had greater resistance to anoikis than control cells (Fig. 3H). Because a link between an increased glycolytic metabolism and the epithelial-mesenchymal transition has been established [4], we measured the glycolytic activity in the control, Serpine1ATG* and Serpine1wt cells using the Seahorse Analyzer, and we found that the upregulation of Serpine1ATG* mRNA significantly increases basal and compensatory glycolysis (Fig. 3I). Altogether, these results indicate that the overexpression per se of mRNA *Serpine1* confers mesenchymal characteristics to NMuMG cells.

### *Serpine1* mRNA increased TRA2B protein level without affecting *Tra2b* mRNA expression level

**Fig. 4:**
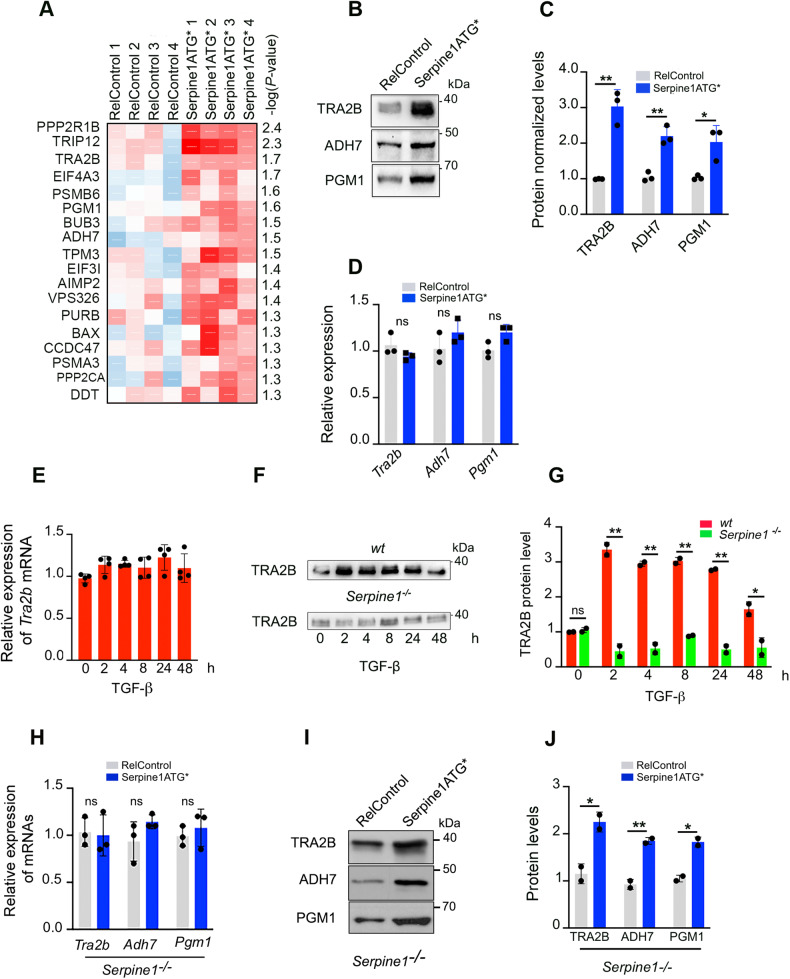
*Serpine1* mRNA upregulation increases levels of specific proteins. **A** Heatmap of upregulated proteins detected at significantly different levels (false discovery rate: <0.05) in Serpine1ATG cells versus RelControl cells. **B** Representative immunoblotting of TRA2B, ADH7 and PGM1 proteins in RelControl and Serpine1ATG* cells. Protein-loading normalization was performed by measuring total protein directly on the membrane using the criterion stain-free gel imaging system. **C** Quantification of TRA2B, ADH7, and PGM1 in three independent experiments. Error bars represent S.D. **P < 0.01, *P < 0.05 by two-tailed Student’s *t* test. **D** *Tra2b, Adh7*, and *Pgm1* mRNAs were similarly expressed by RelControl and Serpine1ATG* cells. Expression was normalized to *Hprt* mRNA. Experiments were performed independently three times; error bars represent S.D. ns not significant. **E** Relative levels of *Tra2b* mRNA in cells treated with TGF-β at the indicated times. **F** Representative immunoblotting of TRA2B in control and *Serpine1*-depleted cells treated with TGF-β at the indicated times. **G** Quantification of TRA2B in two independent experiments. Error bars represent S.D. **P < 0.01, *P < 0.05, ns not significant by two-tailed Student’s *t* test. **H** *Tra2b, Adh7*, and *Pgm1* mRNAs were similarly expressed in *Serpine1*-depleted control cells and *Serpine1*-depleted cells expressing Serpine1ATG* mRNA. Expression was normalized to *Hprt* mRNA; error bars represent S.D. ns not significant. **I** immunoblotting of TRA2B, ADH7 and PGM1 proteins in *Serpine1*-depleted control cells and *Serpine1*-depleted cells expressing Serpine1ATG* mRNA. Protein-loading normalization was performed by measuring total protein directly on the membrane using the criterion stain-free gel imaging system. **J** Quantification of TRA2B, ADH7, and PGM1 proteins in two independent experiments. Error bars represent S.D. **P < 0.01, *P < 0.05 by two-tailed Student’s *t* test.

iCLIP results were analyzed, and sequences corresponding to crosslinked sites were obtained to identify *Serpine1*-associated miRNAs. These sequences comprised 20 bp upstream and 20 bp downstream of the crosslinking site. Once these sequences were determined, 31 were selected because they were specific to TGF-β treatment; that is, they did not overlap with any other sequence corresponding to the control conditions (Supplementary Table S5). One hundred eighty-nine miRNAs seed sequences were contained in the selected sequences according to the *miRBase database (*https://www.mirbase.org/*)* (Supplementary Table S6). Thus, after four h of treatment with TGF-β, *Serpine1* mRNA was the AGO2-associated transcript with the highest abundance of miRNA seeds per kb of the transcriptome (Supplementary Fig. S4). Nine of these seed sequences were selected (Table 3) after discarding those miRNAs that were absent or minimally represented in NMuMG cells [5]. To determine the proteins whose expression levels were affected by the expression of *Serpine1* mRNA, we performed mass spectrometry and analyzed the proteomic profile of Serpine1ATG* and RelControl cells. A total of 18 proteins were found to be significantly increased by Serpine1ATG* expression (Fig. 4A). The increased expression level of proteins TRA2B, ADH7, and PGM1 was confirmed by western blot (Fig. 4B, C). Likewise, it was determined that these genes were not affected transcriptionally by Serpine1ATG* overexpression (Fig. 4D). We focused on the TRA2B splicing factor due to its relevance in tumorigenesis and metastasis [6, 7]. A time course of mRNA *Tra2b* expression after TGF-β treatment showed that *Tra2b* mRNA levels were hardly affected (Fig. 4E); however, TRA2B protein levels increased significantly after two hours of treatment, confirming its rapid post-transcriptional regulation (Fig. 4F, G). *Serpine1-*depleted cells were generated by CRISPR-Cas9 technology to verify that the increase of TRA2B protein level was an effect of *Serpine1* mRNA. NMuMG cells were edited by CRISPR using two pairs of single guide RNAs (sgRNAs) located in the promoter and second exon of *Serpine1*, respectively, obtaining a deletion of 2941 bp (Supplementary Fig. S5A, B). Complete inhibition of *Serpine1* mRNA was achieved in homozygous clones (Clones 2 and 6) even after TGF-β treatment, as shown by qPCR analysis (Supplementary Fig. S5C, D). TRA2B protein levels were not affected in *Serpine1*-depleted cells after TGF-β treatment for the short or long term (Fig. 4F, G), indicating the relevant role of *Serpine1* mRNA in the post-transcriptional regulation of *Tra2b*.

**Table 3:** Location of selected miRNAs recognition elements in *Serpine1* mRNA.

miRNA	Location in mRNA Serpine1
miR-128-3p	3’UTR
miR-130-5p	Exon 3 (1), 3’UTR (2)
miR-149-5p	Exon 3
miR-185-5p	Exon 2
miR-1943-5p	3’UTR
miR-210-5p	Exon 7
miR-296-3p	Exon 6
miR-30a/d/e/-3p	3’UTR
miR-30b-3p	3’UTR
miR-331-3p	3’UTR
miR-350-3p	Exon 1
miR-532-5p	3’UTR
miR-6539	3’UTR
miR-7a-5p	Exon 7
miR-93-3p	Exon 2

It should also be noted that the *Tra2b, Adh7*, and *Pgm1* mRNA levels did not show significant changes in *Serpine1-*depleted cells compared to RelControl cells (Fig. 4H). However, TRA2B, ADH7, and TRA2B protein levels were significantly increased in *Serpine1-*depleted cells by expression of Serpine1ATG* (Fig. 4I, J). *Serpine1-*depleted cells also showed a notably lower migratory capacity than Relcontrol cells (Supplementary Fig. 6A, B and Supplementary Movie S2). However, this migratory capacity increased when Serpine1wt or Serpine1ATG* were overexpressed (Supplementary Fig. 6C and D and Supplementary Movie S3). Collectively, these results emphasized that *Serpine1* mRNA acts per se as a post-transcriptional regulator in addition to encoding the SERPINE1 protein.

### *Serpine1* mRNA sequesters miR-130b-5p to regulate the expression of TRA2B splicing factor

**Fig. 5:**
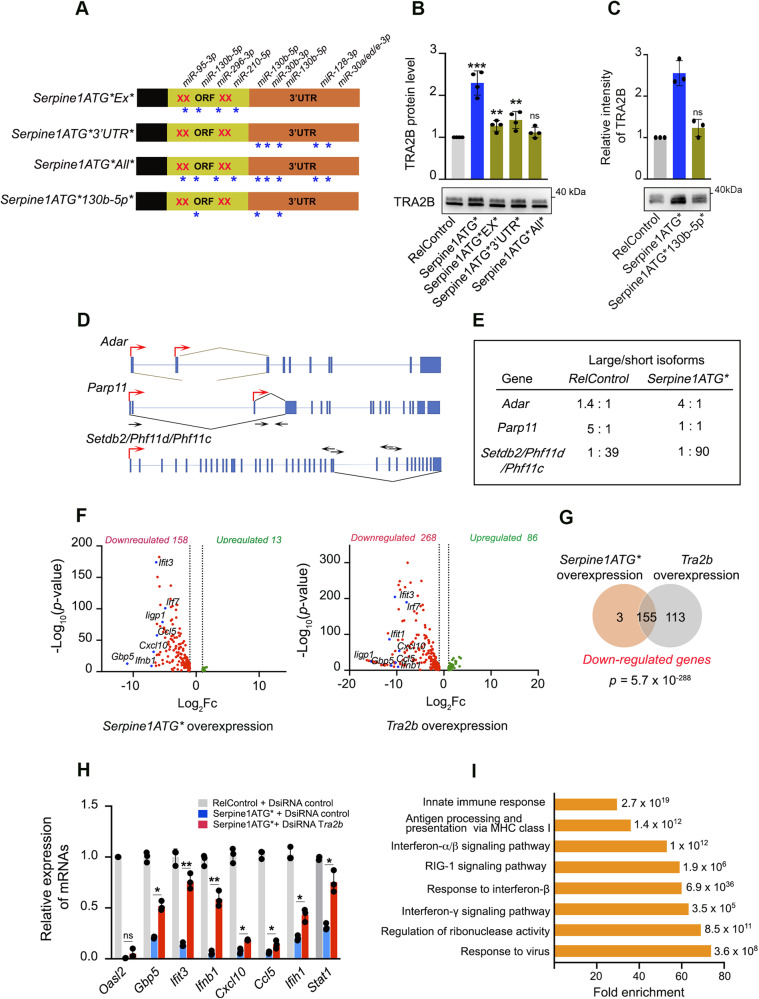
*Serpine1* mRNA and TRA2B mediate the downregulation of innate immune system genes. **A** Diagram of Serpine1 constructs containing the Serpine1 cDNA with selected miRNAs sites in the coding region mutated (Serpine1ATG*Ex*); the cDNA with selected miRNAs sites in the 3’UTR mutated (Serpine1ATG*3’UTR*); the cDNA with selected miRNAs sites in both coding region and 3’UTR mutated (Serpine1ATG*All*); and the cDNA with all three miR-130b-5p sites mutated (Serpine1ATG*130b-5p*). ORF, open reading frame, Xs represent mutations of the ATG codons, *represent mutations of the selected miRNAs sites in the Serpine1 cDNA. **B** Quantification and representative immunoblotting of TRA2B protein in RelControl, Serpine1ATG*, Serpine1ATG*Ex*, Serpine1ATG*3’UTR* and Serpine1ATG*All* cells. **C** Quantification and representative immunoblotting of TRA2B protein in RelControl, Serpine1ATG* and Serpine1ATG*130b-5p* cells. **D** Cartoon illustrating the splice junctions of transcripts differentially spliced between RelControl and Serpine1ATG* or Tra2b cells. Each gene is diagrammed by vertical blue bars (exons) and thin horizontal lines (introns) with black arrows representing specific RT-PCR primers and red arrows start transcription sites. **E** Regulated splicing in RelControl and Serpine1ATG* cells was analyzed by RT-qPCR, and the rate of isoforms was indicated. **F** Volcano plot for differentially expressed genes in RelControl versus Serpine1ATG*-overexpression cells and RelControl versus *Tra2b*-overexpression cells. Genes upregulated and downregulated with more than 2-fold change with a *P*-value < 0.05 are depicted in green and red boxes respectively. **G** Venn diagram showing the intersection of genes downregulated in Serpine1ATG*- and Tra2b-overexpression cells. The probability of overlapping based on hypergeometric distribution is provided. (H) Relative mRNA quantification of indicated genes by qPCR in RelControl, Serpine1ATG*, and *Tra2b* DsiRNA-Serpine1ATG* cells. For each mRNA, results are presented relative to the average value of *Hprt* reference gene. Error bars represent S.D. **P < 0.01, *P < 0.05, ns not significant by two-tailed Student’s *t* test. **I** Gene set enrichment analysis of downregulated genes in Serpine1ATG*- and *Tra2b*-overexpression cells.

The binding sites of the selected miRNAs that interact with the *Serpine1* mRNA are all present in the *Tra2b* mRNA sequence. Three constructs were generated in the pSINDUAL lentiviral vector to determine which of them could be involved in the post-transcriptional regulation of *Tra2b*; one of them, Serpine1ATG*Ex*, contained mutated binding sites located in the exons (miR-93-3p, miR-130b-5p, miR-296-3p, and miR-210-5p), another construct, Serpine1ATG*3’UTR*, had mutated binding sites located in the 3’UTR region (miR-130b-5p (2 binding sites), miR-30b-3p, miR-128-3p and miR-301/e/d-3p), and another one, Serpine1ATG*All*, contained mutated all binding sites (Fig. 5A). The overexpression of the three constructs decreased the expression levels of the TRA2B protein (Fig. 5B). We focused on miR-130b-5p, which had three binding sites in the *Serpine1* mRNA, one in exon three and two in the 3’UTR region. Cells overexpressing *Serpine1* mRNA with the mutated miR-130b-5p sites showed markedly lower levels of TRA2B protein, indicating that this miRNA was involved in the post-transcriptional regulation of *Tra2b* (Fig. 5C).

### *Serpine1* mRNA and TRA2B protein expression downregulate genes involved in the immune response

Since the TRA2B splicing factor expression level was increased by *Serpine1* RNA overexpression, we analyzed and compared the alternative splicing events produced by these two conditions. The overexpression of the *Serpine1* mRNA and TRA2B protein induced 16 and 21 differential events of AS, respectively (Supplementary Tables S7 and S8). Three of these events, affecting the genes *Adar1, Parp11*, and the *Phf11d/Phf11c/Setdb2* precursor transcript, were common and were validated by RT-qPCR. Two isoforms of *Adar1* were detected, increasing the isoform that excluded exons 2 and 3. In the case of *Parp11*, the isoform that excluded exon1 was enriched, and in the case of the *Phf11d/Phf11c/Setdb2* precursor RNA, the short isoform lacking exons 27 to 38, which corresponds to the *Phf11d* gene, was enriched (Fig. 5D, E). We next reasoned that analysis and comparison of global gene expression patterns in *Serpine1*- and *Tra2b*-overexpressing cells could reveal further clues toward elucidating their function. RNA-seq analysis examined the global gene expression profile of RelControl, Serpine1ATG*, and *Tra2b*-overexpressing cells (Supplementary Tables S9 and S10). 13 upregulated and 158 downregulated genes were identified with a *P* < 0.05 and a fold change (FC) > 2.0 in the Serpine1ATG* cells compared to RelControl cells. In *Tra2b*-overexpressing cells, 86 upregulated and 268 downregulated genes were identified compared to RelControl cells (Fig. 5F). Analysis of the downregulated genes showed that 98% of the genes downregulated by Serpine1ATG* overexpression were also downregulated by *Tra2b* overexpression (Fig. 5G), suggesting that most of the changes produced by the expression of *Serpine1* mRNA were carried out through the splicing factor TRA2B. To further confirm the role of splicing factor TRA2B as a downstream effector of *Serpine1* mRNA overexpression, we used *Tra2b*-DsiRNA and *control*-DsiRNA. We determined the expression levels of 8 downregulated selected genes in Serpine1ATG*-expressing cells. We found expression partial reversion of the selected genes *Oasl2, Gbp5, Ifit3, Ifnb1, Cxcl10, Ccl5, Ifih1* and *Stat1* in *Tra2B*-DsiRNA compared to DsiRNA-control cells (Fig. 5H). Increased expression levels of TRA2B protein and downregulation of the selected genes were also confirmed in immortalized human epithelial cell line hTERT RPE1 expressing *Serpine1* mRNA (Supplementary Fig. S7A, B). Gene ontology of genes downregulated by mRNA *Serpine1* and TRA2B expression showed an outstanding enrichment in the regulation of innate immune response, type I interferon signaling pathway, response to interferon beta, interferon-gamma-mediated signaling pathway and antigen processing, and presentation of peptide antigen via MHC class I. Similar results were found in KEGG and Reactome enrichment analyses (Fig. 5I and Supplementary Tables S9 and S10).

### High levels of *SERPINE1* mRNA correlate with low levels of T cell infiltration in colon adenocarcinomas

**Fig. 6:**
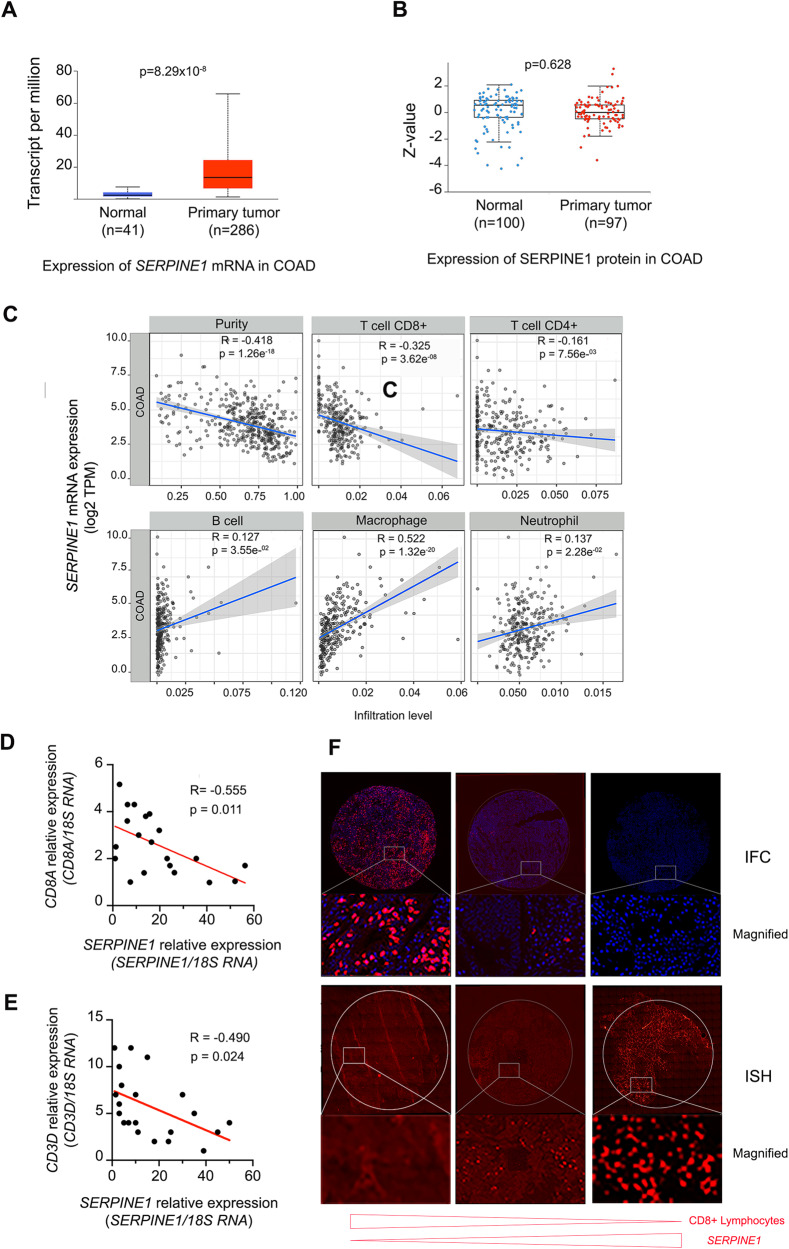
*SERPINE1* mRNA expression is associated with the downregulation of genes involved in different pathways that regulate the innate immune response and decrease infiltrated lymphocytes in human colon adenocarcinomas. **A**, **B** Expression of *SERPINE1* mRNA and SERPINE1 protein in COAD samples and adjacent normal samples from TCGA and CPTAC databases respectively. **C** The Spearman correlation coefficients in the analysis of the expression levels of *SERPINE1* mRNA and the infiltration levels of CD8+ T cells, CD4 + T cells, B cells, macrophages and neutrophils in patients with COAD (*n* = 458). **D**, **E** Linear correlation analysis between *SERPINE1* and *CD8A* mRNAs and *SERPINE1* and *CD3A* mRNAs, respectively, in COAD surgical samples. **F** Representative images of COAD samples showing low, medium, and high signal for *SERPINE1* mRNA, detected by in situ hybridization (ISH) and the corresponding immunofluorescence signal (IFC) using anti-CD8A antibody (red) and DAPI (blue).

Bioinformatics tools have provided a preliminary understanding of *SERPINE1* expression and prognosis in different tumors. According to a pan-cancer data set of TGCA, expression levels of *SERPINE1* mRNA were significantly increased (*p* < 0.001) in breast invasive carcinoma (BRCA), colon adenocarcinoma (COAD), stomach adenocarcinoma (STAD), glioblastoma multiforme (GBM), kidney renal clear cell carcinoma (KIRC), and head and neck squamous cell carcinoma (HNSC). However, *SERPINE1* mRNA expression was significantly lower in Kidney chromophobe (KICH), Liver hepatocellular carcinoma (LIHC), and Skin cutaneous melanoma (SKCM) compared with adjacent normal tissues (Supplementary Fig. 8A). Paradoxically, SERPINE1 protein levels were not associated with *SERPINE1* mRNA levels in some tumors such as COAD, where protein levels were even lower in tumor tissue than in adjacent normal tissue (Supplementary Fig. 8B and Fig. 6A, B) [8, 9]. Therefore, this tumor’s significantly poorer survival associated with high SERPINE1 levels (Supplementary Fig. 8C) could be attributed to elevated mRNA *SERPINE1* levels and the downregulation of immune response-associated genes. We used the TIMER2.0 database [10] to investigate the correlation in COAD between *SERPINE1* expression and B cells, CD8+ T cells, CD4 + T cells, macrophages, and neutrophils infiltrations levels. CD8+ and CD4 + T cells show an inverse correlation with *SERPINE1* mRNA levels (R = −0.325, *P* < 0.001. and R = −0.161, *P* < 0.01 respectively), and B cells, macrophages, and neutrophils a positive correlation (R = 0.127, *P* < 0.05, R = 0.522, *P* < 0.001 and R = 0.137, *P* < 0.05 respectively) (Fig. 6C). Given the critical role of CD8+ T cells in immune-mediated tumor rejection and as a prognosis factor in many solid tumors, we next determined, using RT-qPCR analysis, the levels of *SERPINE1* and *CD8A*, a cell surface receptor found on cytotoxic T lymphocytes, in 20 surgical COAD specimens. A matched scatter plot revealed a consistent inverse correlation between *SERPINE1* and *CD8A* mRNA expression levels (R = −0.555, *P* < 0.011), in concordance with results obtained from the tumor immune estimation resource (Fig. 6D). Likewise, inverse correlation (R = −0.490, *P* < 0.024) was also found between levels of *SERPINE1* and *CD3D* mRNA, a marker for total lymphocytes (Fig. 6E). *SERPINE1* mRNA levels and the number of infiltrated lymphocytes were also analyzed in COAD surgical samples by *SERPINE1* mRNA in situ hybridization and CD8A protein immunofluorescence (Fig. 6F), confirming the inverse correlation determined by RT-qPCR analysis.

## Discussion

In this work, we have studied the post-transcriptional regulation of EMT by analyzing RNAs associated with the RISC complex early. The most enriched RNA in the RISC complex was the S*erpine1* mRNA, which paradoxically is one of the strongly induced mRNAs at the beginning of the EMT process. This study demonstrates that *Serpine1* mRNA exhibits a non-coding function in EMT by sequestering miRNAs, unlocking the translation of different mRNAs, in addition to encoding the SERPINE1 protein.

Analysis of iCLIPseq data from cells treated with TGF-β for 4 and 48 h revealed enrichment of numerous RNAs (mRNAs and lncRNAs) in the RISC complex, suggesting their potential role as miRNAs sponges. This additional post-transcriptional gene regulation mechanism may be common in other biological processes [11, 12]. The distribution of iCLIPseq reads was very similar in the conditions analyzed. We also observed that the interaction between the RNAs and the RISC complex occurs mainly and in very similar proportions in the coding and 3’UTR regions. This suggests that miRNA binding sites in coding regions are more widespread than previously considered and nearly as prevalent as in 3’UTRs. This finding supports the hypothesis that the coding regions may contain additional information besides the amino acid sequence of the encoded protein [13, 14].

*Serpine1* mRNA expression was studied by mutating two contiguous ATG codons at the TSS and two other adjacent codons 800 nt downstream of the TSS to prevent its translation. The expression of *Serpine1* mRNA conferred mesenchymal characteristics to the cells, such as increased migratory and invasive capacity, resistance to anoikis, and increased glycolytic activity. These abilities have been associated with SERPINE1 protein overexpression [15–17]. Our study underlines the importance of dissecting the effects of the mRNA molecule per se and not only those due to the encoded protein. In many studies that correlate the expression of the *Serpine1* gene with tumorigenesis, the methodology used does not differentiate between the effects caused by the mRNA and the protein [18, 19]. As transcriptomes are easier to quantify than proteomes, mRNA abundance is often used as a proxy for protein abundance. However, large-scale studies in humans and model organisms have shown that there is only a moderate correlation between mRNA and protein levels for most genes [20, 21]. It is important to note that most studies on tumors consider mRNA levels and not protein levels. Analysis of SERPINE1 protein levels in 20 different tumor types collected in The Human Protein Atlas database reveals that protein levels are undetectable or extremely low. The UALCAN platform enables the analysis of protein levels in various tumors through the CPTAC application (Clinical Proteomic Tumor Analysis Consortium). According to the platform, breast, colon, ovarian, and lung tumors that express high levels of *SERPINE1* mRNA do not show significant changes in SERPINE1 protein levels in tumor tissue and adjacent normal tissue. This suggests that the tumorigenic capacity of *SERPINE1* mRNA is, at least in large part, associated with its non-coding function. However, in tumors such as head and neck carcinoma, kidney renal clear cell carcinoma, and glioblastoma with high levels of both mRNA *Serpine1* and protein SERPINE1, both could contribute to the migration, invasiveness, and resistance to anoikis of the tumor cell through different mechanisms.

In cells overexpressing *Serpine1* RNA, TRA2B is one of the proteins that markedly increases its expression level without affecting *Tra2b* mRNA levels. TGFβ treatment of *Serpine1*-depleted cells confirmed that the increase in TRA2B depends on *Serpine1* mRNA expression. miR-130b-5p regulates *Tra2b* post-transcriptionally through three interaction sites: one in the coding region and two in the 3’UTR region. Several cancers, including breast, cervical, ovarian, and colon, show an increase in TRA2B protein levels [6]. *TRA2B* upregulation is associated with invasive breast cancer, and medium to high *TRA2B* expression correlates with a poorer prognosis [7, 22]. The molecular mechanisms through which TRA2B levels are altered in tumors remain poorly unknown. Besides *TRA2B* gene amplification [23], increased levels of the ETS-1 and c-MYC transcription factors have been proposed as possible mechanisms to explain *TRA2B* upregulation in cancer cells [7, 24]. Our findings add another potential mechanism by which TRA2B protein levels would increase in tumors overexpressing *SERPINE1* mRNA without being *TRA2B* gene transcriptionally affected. Furthermore, we demonstrate that expression of *Serpine1* and *Tra2b* produce very similar gene expression changes, with 98% of genes downregulated by *Serpine1* being downregulated by *Tra2b*. Since the downregulation of genes by *Serpine1* expression was reversed by lowering *Tra2b* levels, we conclude that most of the expression changes caused by *Serpine1* depend on *Tra2b*.

According to the EPIC (Estimating the Proportions of Immune and Cancer cells) immune deconvolution method, *SERPINE1* mRNA expression negatively correlates with CD8+ T cell infiltration level in diverse cancer types. In agreement with these data, we show that mRNA levels of *SERPINE1* inversely correlate with the levels of specific lymphocyte markers CD8A and CD3 in surgical samples of colon tumors. The inverse correlation was also observed by combining in situ hybridization of mRNA *SERPINE1* and immunofluorescence of the lymphocyte marker CD8A. The immune system plays a crucial role in the eradication of tumor cells. Notably, the presence of tumor-infiltrating lymphocytes is associated with favorable long-term outcomes in ovarian cancer [25, 26], colon cancer [27], and most other solid tumor types [28]. Several studies have found a strong correlation between the expression of CCL5 and CXCL10 in tumors and the infiltration of CD8+ T lymphocytes in different types of cancer, such as colorectal cancer [29, 30], melanoma [31], and esophageal squamous cell carcinoma [32]. Here, we show that the expression of *Serpine1* and *Tra2β* downregulates the expression of the chemokines *Ccl5* and *Cxcl10*, as well as the expression of the genes Mad5, Zap, Rig1, and Zbp1, which act as cytosolic sensors of nucleic acids, and the major histocompatibility complex class I (MHC-I) molecules H2-K1 and H2-K2. After forming protein-nucleic acid complexes, DNA and RNA sensor molecules recruit and activate TANK-binding kinases-1 (TBK1) and interferon regulatory factors (IRFs), which finally initiate the transcription of *IFN-1* and inflammatory cytokines [33–35]. Furthermore, one primary mechanism through which solid tumors can avoid anti-tumor immunity is the downregulation of MHC-I, which causes reduced recognition by CD8+ T-cells [36, 37]. According to the EPIC (Estimating the Proportions of Immune and Cancer cells) immune deconvolution method, *SERPINE1* mRNA expression negatively correlates with CD8+ T cell infiltration level in diverse cancer types. In agreement with these data, we show that mRNA levels of *SERPINE1* inversely correlate with the levels of specific lymphocyte markers CD8A and CD3 in surgical samples of colon tumors. The inverse correlation was also observed by combining in situ hybridization of mRNA *SERPINE1* and immunofluorescence of the lymphocyte marker CD8A. The immune system plays a crucial role in the elimination of tumor cells. Notably, the presence of tumor-infiltrating lymphocytes is associated with favorable long-term outcomes in ovarian cancer [25, 26], colon cancer [27], and most other solid tumor types [28]. Several studies have found a strong correlation between the expression of CCL5 and CXCL10 in tumors and the infiltration of CD8+ T lymphocytes in different types of cancer, such as colorectal cancer [29, 30], melanoma [31], and esophageal squamous cell carcinoma [32]. Here, we show that the expression of *Serpine1* and *Tra2b* downregulates the expression of the chemokines *Ccl5* and *Cxcl10*, as well as the expression of the proteins MAD5, ZAP, RIG1, and ZBP1, which act as cytosolic sensors of nucleic acids, and the major histocompatibility complex class I (MHC-I) molecules H2-K1 and H2-K2. After forming protein-nucleic acid complexes, DNA and RNA sensor molecules recruit and activate TANK-binding kinases-1 (TBK1) and interferon regulatory factors (IRFs), which finally initiate the transcription of *IFN-1* and inflammatory cytokines [33–35]. Furthermore, one primary mechanism through which solid tumors can avoid antitumor immunity is the downregulation of MHC-I, which causes reduced recognition by CD8+ T-cells [36, 37]. Therefore, the downregulation of these factors, caused by *Serpine1* and *Tra2b* expression, would lead to fewer CD8+ T cells in tumors, promoting immune evasion.

SERPINE1 is an essential element of the fibrinolytic system, acting as an anti-fibrinolytic molecule in the normal coagulation process, blocking extracellular matrix degradation and tissue remodeling. This function suggests that it may have an antitumorigenic function. However, *SERPINE1* has a paradoxical pro-tumorigenic role, promoting tumor cell survival and metastasis. Small molecules and antibodies inhibitors against SERPINE1, originally developed as antithrombotic agents, have shown efficacy in models of acute thrombosis by promoting rapid thrombus re-permeabilization [38, 39]. However, there is no evidence that inhibition of SERPINE1 could have any therapeutic effect in tumor treatment [40–42]. The data presented here is the first clear evidence that a protein-coding gene, *Serpine1*, operates largely independently of its protein-coding function as a competing endogenous RNA conferring mesenchymal features to the cell. As a non-coding RNA, it sequesters miR-130b-5p, canceling the repressive activity of this miRNA on *Tra2b* mRNA and downregulating genes involved in the immune response. This dual function of *SERPINE1* mRNA clarifies the paradox between the expected antitumor effect of *SERPINE1* as a serine protease inhibitor and the pro-tumorigenic effect of *SERPINE1* reported by numerous clinical studies. Hence, additional studies on the regulation and function of *SERPINE1* in cancer may provide insight into the molecular mechanisms of tumorigenesis as well as provide therapeutic targets that may help in the treatment of cancer.

## Materials and methods

### Cell culture

Normal Mouse Mammary Gland NMuMG epithelial cells were grown in DMEM supplemented with 10% fetal bovine serum FBS), 2 mM L-glutamine, 1% penicillin-streptomycin (Sigma-Aldrich, St Louis, MO, USA), and 10 µg/mL insulin (Sigma). Immortalized human retinal pigment epithelial cells hTer-RPE1 (Clontech, Mountain View, CA, USA) were maintained in DMEM/F12 supplemented with 10% FBS, 2 mM L-glutamine, and 1% penicillin-streptomycin (Sigma). Cells were grown at 37 °C under a humidified atmosphere with 5% CO_2_.

### Antibodies and reagents

The following antibodies were used in our study: anti-TRA2B (Santa Cruz; sc166829), anti-PGM1 (Invitrogen; PA5-55008), anti-ADH7 (Invitrogen; PA5-97437), anti-mouse Serpine1 (R&D Systems; AF3828), anti-human Serpine1 (ThermoFisher, #66261-1-IG), anti-CD8A (Sigma-Aldrich, HPA037756), anti-AGO2 (Wako Chemicals; clone 2D4, # 018-22021). TGF-β was purchased from PeproTech (London, UK).

### Transfections, lentivirus production, and infection assays

For lentiviral production, 2.8 × 10^6^ HEK293T cells were seeded onto a 10-cm Petri dish and transfected with Lipofectamine 2000 (Invitrogen, Life Technologies, Karlsruhe, Germany) using 15 µg of the transfer vectors pHRSIN-DUAL with 10 and 5 µg of the HIV packaging plasmids pCMVDR8.91 and pVSVG respectively. Lentiviruses were harvested 48 h post-transfection, passed through a 0.45-µm filter, and concentrated by ultracentrifugation at 100,000 g for 90 min. Virus particles were resuspended in serum-free DMEM and stored at −80 °C. Titers of pHRSIN-DUAL lentiviral particles were determined by flow cytometry of NMuMG-infected cells. For ectopic expression of Serpine1 (with the different mutations), NMuMG cells were infected with lentiviral supernatants (MOI 2) containing 4 µg/mL polybrene. The dual-promoter lentivector pHRSIN-DUAL (also known as pHRSIN-CSGWdINotI_pUb_Em) was kindly provided by Mary K. Collins (Queen Mary’s Blizard Institute, London).

### RNA interference

A TriFECTa DsiRNA kit (mm.Ri.Tra2b.13) containing one non-targeting control DsiRNA (DS NC1), one transfection control DsiRNA (TYE 563DS), one positive control DsiRNA (HPRT-S1 DS) and three DsiRNA targeting *Tra2b* mRNA (mm.Ri.Tra2b.13.1, mm.Ri.Tra2b.13.2, mm.Ri.Tra2b.13.3) were purchased from Integrated DNA Technologies (IDT). The Tra2b targeting sequences are shown in Supplementary Table S11. Cells were transfected with RNA duplexes using Viromer (*Lipocalix*, Halle, Sachsen‐Anhalt, Germany) following the manufacturer’s protocol TriFECTa DsiRNA Kit.

### RNA isolation, and real-time PCR analysis

Total RNA was isolated using the TRIzol reagent according to the manufacturer’s instructions (Invitrogen). Total RNA was treated with Turbo DNase (Ambion) and reverse transcribed using a mix of oligo(dT)_20_ and random hexamers and SuperScript III reverse transcriptase (Invitrogen). Real-time quantitative PCR (qPCR) was performed using iTaq Universal SYBR Green Supermix (Bio-RAD) and analyzed on an Applied Biosystems 7500 Fast-Real-Time PCR System. Expression levels were normalized to HPRT levels. For RNA isolation from tumor samples, frozen samples were cut into small pieces and placed in TRIzol Reagent (Invitrogen). Next, they were mechanically homogenized using a Pro250 homogenizer (PROScientific, Oxford, CT, USA). Total RNA was extracted according to the manufacturer’s instructions.

### CRISPR–Cas9-mediated genetic cell engineering

The Alt-R CRISPR-Cas9 System (IDT) was used to generate *Serpine1*-depleted NMuMG cells, according to the manufacturer’s protocol. Upstream and downstream guides were designed within (−600/−500 bp) and (+1000/+1100) of the TSS. Guide sequences are listed in Supplementary Table S11. Successfully transfected cells were sorted by fluorescent-activated cell sorting (FACS) and individually distributed into 96-well plates. After 10–14 days, cells were plated into two separate 96-well flat-bottom plates. One was kept to allow clones to grow, and the other was to screen each clone for the designed deletion. Genomic DNA was isolated using the Purelink Genomic DNA Kit (Invitrogen) according to the manufacturer’s protocol. Deletions were analyzed by PCR, and deletions amplicons were sequenced to identify the precise deletion. RNA was isolated from monoallelic and biallelic deletion clones and analyzed by RT-qPCR to quantify the *Serpine1* mRNA expression.

### RNA-seq analysis

Total RNA was isolated using TRIzol (Invitrogen) and depleted of DNA by DNase treatment (TURBO DNase, Invitrogen). Libraries were prepared with the TruSeq Stranded mRNA kit (Illumina, San Diego, CA, USA) and sequencing was performed with a Novaseq system (Illumina) with 75 bp single-end reads with the Genomic Unit of CABIMER (Sevilla, Spain). Two biological replicates for each condition were sequenced. Reads were aligned to mouse genomes (GRCh38/hg38) using the subjunc function from the Rsubread package. The downstream analysis was performed on .bam files with duplicates removed using the samtools (v0.1.19) rmdup command. Differential gene expression analysis and statistics were performed using the DESeq2 Bioconductor. Differentially expressed genes with *p-*adjusted values < 0.05 and Log_2_FC > 0.5 (upregulated genes) or Log_2_FC < −0.5 (downregulated genes) were selected for further analysis. Gene ontology (GO) functional categories were analyzed using DAVID Bioinformatics Resources. Non-adjusted *p* values were used to determine the enrichment significance.

### iCLIP

iCLIP was performed as described [43]. NMuMG cells were subjected to 100 mJ/cm^2^ of UV-C irradiation to covalently crosslink proteins to nucleic acids, in vivo. RNase I (1:160) was used to partially fragment RNA during cell lysis. AGO2–RNA complexes were immunopurified using an immobilized antibody on immunoglobulin G–coated magnetic beads. RNAs were then ligated to an RNA adapter at their 3’ ends and radioactively labeled for visualization purposes. Denaturing gel electrophoresis was performed to remove RNAs not covalently linked to the protein, then transferred to a nitrocellulose membrane. RNA was recovered from the membrane through proteinase K digestion. Reverse transcription oligonucleotides were designed to have two inversely oriented adapter regions separated by a BamHI restriction site and a barcode region to mark the experiment and the individual cDNA molecules at their 5’ ends. The cDNA molecules were size-purified using denaturing gel electrophoresis, circularized, and cut between the two adapter regions by BamHI. The linearized cDNAs were then PCR-amplified using primers complementary to the adapter regions and were subjected to high-throughput sequencing. The oligonucleotide sequences are listed in Supplementary Table S11.

### iCLIP bioinformatics analysis

For iCLIP-seq analysis, first reads from the three independent experiments were filtered using the FASTQ Toolkit program. Then, reads were aligned to the mouse transcriptome using the gencode.vM1.annotation.gtf for mm9. Transcripts were filtered to use only the longest transcripts as described [44]. *Getfasta* from *Bedtools* was used to obtain the longest FASTA format transcripts. Reads were then aligned to transcripts using con bowtie (v 2.1.0). To calculate the correlation between the replicates, the R/Bioconductor DESeq2 package (1.18.1) was used, quantifying the number of reads per gene in each condition and performing a principal component analysis (PCA). After verification of the high correlation between replicates, alignment files of the three replicates from each condition were merged using *samtools*. For the detection of crosslinking sites, *PureCLIP* (1.1.1) was used by using the -nim 4 parameter. 2572 binding sites were found for the control condition, 6760 for TGFβ4 h, and 4114 for TGFβ48 h. Once identified, the size of these binding sites was lengthened according to the center of the binding site +/−20 bp. These new 40 bp regions centered on the binding site were called binding regions and were used to identify binding motifs. Alignment files for each condition were filtered to obtain the reads that match the binding regions for each gene using bedtools intersect. Once the bam files were filtered, *samtools idxstats* was used to calculate the reads contained in each gene. Identification of miRNA binding motifs in the binding regions was performed as described [45]. First, we downloaded the mouse miRNA seeds from the miRNA database (http://www.mirbase.org/). These sequences were then processed, obtaining their complementary reverse and creating the “motif_file” using seq2profile.pl script from HOMER software [46]. To search for the representation of the miRNAs seeds in the binding regions identified by iCLIP, “motif_file” was transformed to the format accepted by the MEME suite using the script *motif2meme.pl* (https://gist.github.com/rtraborn/e395776b965398c54c4d). After this, the FIMO program (incorporated in MEME) [47] was executed with the default parameters to search for matches between the miRNAs seeds and the sequences included in the binding regions.

### Western blot analysis

Protein was extracted with RIPA buffer supplemented with phosphatase and protease inhibitors (Roche, Indianapolis, IN, USA), and protein concentration was spectrophotometrically measured by Bradford assay kit (Bio-Rad) according to manufacturer’s protocol. Then, 30–40 µg of protein were resolved by SDS-PAGE and transferred to Immobilon^®^-P PVDF membrane (Merck Millipore, Billerica, MA, USA) according to standard protocols. Membranes were incubated with primary antibody overnight at 4 °C, then washed and incubated with horseradish peroxidase (HRP)-conjugated anti-rabbit IgG or anti-mouse IgG secondary antibodies (Amersham Biosciences) for 1 h at room temperature. Signals were detected with an ECL kit (GE Healthcare, Buckinghamshire, UK) and visualized and quantified using ChemiDoc MP Imaging system (Bio-Rad, Hercules, CA, USA). Protein levels were normalized by measuring total protein directly on the membrane using the criterion stain-free gel imaging system (Bio-Rad).

### Protein identification using mass spectrometry

Proteins were in-gel digested as described [48]. Briefly, protein extracts from RelControl and Serpine1ATG* NMuMG cells were subjected to SDS-PAGE gel, and the whole proteome band was subjected to trypsin digestion upon entering the resolving gel. The resulting peptides were analyzed by nanoliquid chromatography coupled to mass spectrometry, using a Q-Exactive HF mass spectrometer (Thermo Fisher Scientific, Waltham, MA, USA), for protein identification and quantification by spectral counting [49]. Peptide identification from MS/MS data was performed using the probability ratio method [50]. False discovery rates (FDR) of peptide identifications were calculated using the refined method [51]; 1% FDR was used as criterion for peptide identification.

### Immunofluorescence microscopy

Tissue sections were first fixed in 4% paraformaldehyde/phosphate-buffered saline (PBS) for 10 min before being permeabilized in 70% ethanol for 1 hour at room temperature. Next, the tissue sections were blocked with 3% BSA in PBS for 1 h and incubated with CD8A primary antibody (1:100) (Cell Signaling Technology, Danvers, MA, USA) at 4 °C overnight. The sections were washed with PBS and incubated with an appropriate fluorochrome-labeled secondary antibody (1:300) at room temperature for 1 h. Finally, the tissue sections were washed with PBS and mounted using ProLong Diamond Antifade Mountant (Invitrogen). Images were acquired in a THUNDER Imager 3D Tissue microscope from Leica microsystems with a 40x HC PL APO NA0.75 using a K5 Leica camera. Images shown were processed with the Small Volume Computational Cleaning Algorithm (SVCC).

### RNA FISH

RNA-FISH followed the manufacturer’s instructions (Biosearch Technologies, Inc., Petaluma, CA). The custom *SERPINE1* RNA-FISH probes consisted of 47 fluorescently Quasar 570-labeled probes complementary to different regions of the human *SERPINE1* mRNA transcript. Each probe was 20 nucleotides in length (Supplementary Table S11). The tissue sections were fixed in 4% formaldehyde for 10 min at room temperature. They were washed twice in PBS and permeabilized in 70% ethanol for 1 hour at room temperature. The sections were washed with buffer A [10% (v/v) formamide in 1× wash buffer A; Biosearch Technologies, Hoddesdon, UK, catalog no. SMF-WA1-60] and incubated with hybridization buffer (Biosearch Technologies, catalog no. SMF-HB1-10) containing 125 nM RNA-FISH probes in the dark within a humidity chamber at 37 °C overnight. The sections were washed with buffer A, stained with DAPI, and washed with buffer B (Biosearch Technologies, catalog no. SMF-WB1-20). Coverslips were mounted on glass slides using Prolong Diamond Antifade Mountant (Invitrogen). Images were acquired as already described.

### Proliferation, migration, and invasion assays

For growth curves, a real-time and label-free xCELLigence System (Roche) was used. For that, cells were harvested and transferred into 16-well E-plates, which contain electrodes integrated into the bottom surfaces of each well to measure cell index based on impedance. 8k, 4k, 2k, and 1k cells were added per well, and the program was set to take readings every 30 min for 95 h. Cell migration was measured using Culture- Inserts (IBIDI, Martinsried, Germany). The Culture-Inserts were transferred to six-well plates, where cells were seeded at a density of 7 ×10^4^/mL in phenol red-free complete medium in each well. After 24 h of incubation, the Culture-Inserts were removed, and a 400 μm cell-free gap was created. Cell migration was observed with a Leica DMI6000 inverted microscope equipped with a Hamamatsu ORCA-ER camera using LEICA N PLAN 10×, /20× /0.25 objectives and recorded every 20 min for 23 h. Image processing was done using the Leica (LAS) and Adobe Photoshop software. Quantification of cell surface was performed using the multiwave length cell-scoring module of Metamorph Offline software and the WimScratch Wound Healing Module (WIMASIS, Munich, Germany) program. Invasion assays were performed using Transwell chambers (24 wells, 8 μM pore size; Cell Biolabs, San Diego, CA, USA) coated with a basement membrane. Subconfluent cells were serum-starved for 24 h. After detachment with trypsin, cells were washed with PBS, resuspended in serum-free medium, and 5 × 10^4^ cells were added to the upper chamber. Complete medium was added to the bottom well of the chamber. The non-migrated cells were removed from the upper face of the filters using cotton swabs, and the migrated cells on the lower face of the filters were fixed with 3.7% formaldehyde, permeabilized with 100% methanol, and stained with 0.05% crystal violet. Images of random fields were captured from each membrane, and the number of invasive cells was counted. The mean of triplicate assays was used for each experimental condition.

### Anoikis assay

Tissue culture dishes (35 mm) were coated with Poly-HEMA 12 mg/mL dissolved in 95% ethanol, allowed to dry under sterile conditions, and washed with PBS and once with DMEM before use. To induce anoikis, 3.5 × 10^5^ cells resuspended in grown medium containing 0.5% methylcellulose (to avoid clumping of cells) were seeded onto poly-HEMA-coated tissue culture plates and incubated for 24 h at 37 °C. Cells were harvested, resuspended in PBS, fixed with ice-cold ethanol (70% final concentration), resuspended in 300 µL solution of 50 μg/mL propidium iodide and 250 μg/mL DNase-free RNase in PBS, and incubated for 30 min at 37 °C. Cells were evaluated using a FACSCalibur™ cytometer for flow cytometric analysis. Cell-cycle distribution and Sub-G_1_ fraction were determined and quantified using the CellQuest-Pro software.

### Seahorse assay

XFe24 Extracellular Flux Analyzer (Agilent, Santa Clara, CA, USA) was used to determine glycolysis metabolism. For that, cells were plated in 24-well (1.5 × 10^4^ cells/well) Seahorse Assay plates, grown in DMEM containing 10% fetal bovine serum, L-glutamine, antibiotics, and insulin (10 µg/mL) and incubated overnight at 37 °C in a 5% CO_2_ humidified, 95% air incubator. The sensor cartridge was hydrated by adding 500 µL of XF Calibrant Solution (Agilent) at 37 °C in a CO_2_-free incubator overnight. GlycoPER was determined using the Agilent Seahorse XF Glycolytic Rate Assay Kit (Agilent, Cat No: 1033344). Data were analyzed by the software Seahorse XFe (Agilent). All measurements were normalized with the total protein concentration on each well. Each assay was run with 6 replicates per each condition.

### Clinical samples

Tumor and adjacent samples were collected from 20 patients who underwent primary surgery for colon adenocarcinoma in Virgen del Rocío Hospital (Sevilla, Spain). All patients included in the study had not received chemotherapy or radiotherapy before resection. The tumor and the adjacent normal tissue samples were embedded in optimal cutting temperature (OCT) compound. RNA quality was confirmed by Agilent bioanalyzer The Medical Ethics Committee of Virgen del Rocío Virgen Macarena Hospitals approved the study, and patients proffered written informed consent for research purposes and publication.

### Statistical analysis

Data were analyzed by one-way ANOVA and Student’s *t* test comparison, using GraphPad Prism 5 (GraphPad Software, La Jolla, CA, USA). Significant *p*-values are indicated with asterisks as follows: *p < 0.05, **p < 0.01, and ***p < 0.001. The probabilities of overlapping genes were calculated using the Keisan Online Calculator’s hypergeometric distribution (https://keisan.casio.com/exec/system).

### Supplementary information

Supplementary information

Supplementary Figure S1

Supplementary Figure S2

Supplementary Figure S3

Supplementary Figure S4

Supplementary Figure S5

Supplementary Figure S6

Supplementary Figure S7

Supplementary Figure S8

Table S1

Table S2

Table S3

Table S4

Table S5

Table S6

Table S7

Table S8

Table S9

Table S10

Table S11

Movie S1

Movie S2

Movie S3

Original Data File

aj-checklist

## Data Availability

NGS data are available at the NCBI Gene Expression Omnibus (GEO) under the accession number GSE221153 without restrictions. GSE221152 and GSE221076 correspond to accession numbers of iCLIP data and RNA-seq data respectively. Publicly available databases used in our analysis included TCGA Research Network (https://www.cancer.gov/tcga), Tumor Immune Estimation Resource (http://timer.cistrome.org/) and MiRbase (http://www.mirbase.org/).
