# Supplementary material for: Serpine1 mRNA confers mesenchymal characteristics to the cell and promotes CD8+ T cells exclusion from colon adenocarcinomas
Source: Cell Death Discov. 2024 Mar 6;10:116. doi: 10.1038/s41420-024-01886-8 (PMC10917750; doi:10.1038/s41420-024-01886-8)
Supplement: Supplementary file 1 — Supplementary information [file 41420_2024_1886_MOESM1_ESM.docx]

**Supplementary information**

**Supplementary Figure S1.** iCLIP-seq replicates were hierarchically clustered according to sample distances using DESeq2, indicating high similarity between replicates.

**Supplementary Figure S2**. RISC binding sites in *Serpine1* transcript identified by iCLIP-seq in control cells and cells treated for 4 hours with TGF-β. Height of the peaks indicates the number of clusters identified within the region**.**

**Supplementary Figure S3**. Real-time monitoring of *in vitro* proliferation of RelControl and Serpine1ATG* (A) and RelControl and Serpine1wt cells (B) using the exCELLigence system. Slope values calculated from xCELLigence real-time measurements between 40 – 80 h are shown.

**Supplementary Figure S4.** Number of miRNA seeds/kb identified around crosslinking sites in AGO2-associated transcripts versus the number of reads associated to the binding sites in the iCLIP-seq experiment. (A) Data for the control conditions and (B) after TGF-β treatment for 4 h. (B) Transcripts with the highest number of miRNA seeds/kb after TGF-β treatment are highlighted in both panels.

**Supplementary Figure S5. Deletion of a *Serpine1* fragment in NMuMG cells and expression profile**. (A) Scheme showing the 2941 bp deleted fragment of *Serpine1* in NMuMG cells using CRISPR-Cas9 technology. Sites of cleavage by Cas9 in the promoter and the second exon regions are indicated; primers (green arrrows) for clone analysis are shown. (B) Representative gel identifying monoallelic (clones 1, 3, 4, 5, 7, 8, 9 and 10) and biallelic (clones 2 and 6) clones. (C) Relative expression levels of *Serpine1* mRNA in different clones were measured by RT-qPCR analysis. (D) Relative expression of *Serpine1* mRNA in control cells and clones 2, 5, 6 and 9 treated with TGF-β for 4 h. For (C) and (D), error bars represent S.D.

**Supplementary Figure S6.** (A) The migratory capacity of wild type and Serpine1**^-/-^** cells was tested in wound-healing assays. Cells were imaged at 10 min intervals for 25h. The frames of the movie at 0, 7 and 12 h are shown. (B) The frames at 7 and 12 h were used to estimate the percentage of surface covered by the cells. Values represent the average (%) of wound closure. Error bars represent S.D. **P*<0.05 by two-tailed Student’s *t*-test. (C) The migratory capacity of Serpine1**^-/-^** cells and Serpine1**^-/-^** cells expressing *Serpine1ATG** or *Serpine1wt* mRNA was tested in wound-healing assays. Cells were imaged at 10 min intervals for 27 h. The frames of the movie at 0, 15 and 26 h are shown. (D) The frames at 15 and 26h were used to estimate the percentage of surface covered by the cells. Values represent the average (%) of wound closure. Error bars represent S.D. ***P*<0.01, ****P*<0.001 by two-tailed Student’s *t*-test.

**Supplementary Figure S7. Expression of *Serpine1ATG** in human RPE1 cells.** (A) Quantification and representative immunoblotting of TRA2B protein in RPE1 RelControl and *Serpine1ATG**-overexpressing RPE1 cells. (B) Relative mRNA quantification of indicated genes by qPCR in RPE1 RelControl and *Serpine1ATG**-overexpressing RPE1 cells. For each mRNA, results are presented relative to the average value of *HPRT* reference gene. Error bars represent S.D. ***P<0.001, ***P*<0.01, **P*<0.05 by two-tailed Student’s *t*-test.

**Supplementary Figure S8. *SERPINE1* expression in tumors at mRNA and protein levels.** (A) The expression profile of *SERPINE1* mRNA in different types of human cancers and their paired normal tissues from TGCA database. The statistical significance computed by the Wilcoxon test is annotated by the number of stars (*P<0.05, **P<0.01, ***P<0.001). The gray columns indicate that data are available for normal tissues. T, tumor; N, adjacent normal tissue; M, metastasis; ACC, Adrenocortical carcinoma; BLCA, Bladder Urothelial Carcinoma; BRCA, Breast carcinoma; CESC, Cervical squamous cell carcinoma and endocervical adenocarcinoma; CHOL, Cholangio carcinoma; COAD, Colon adenocarcinoma; DLBC, Lymphoid Neoplasm Diffuse Large B-cell Lymphoma; ESCA, Esophageal carcinoma; GBM, Glioblastoma multiforme; HNSC, Head and Neck squamous cell carcinoma; KICH, Kidney Chromophobe; KIRC Kidney renal clear cell carcinoma; KIRP, Kidney renal papillary cell carcinoma; LAML, Acute Myeloid Leukemia; LGG, Brain Lower Grade Glioma; LIHC, Liver hepatocellular carcinoma; LUAD, Lung adenocarcinoma; LUSC, Lung squamous cell carcinoma; MESO, Mesothelioma; OV, Ovarian serous cystadenocarcinoma; PAAD, Pancreatic adenocarcinoma; PCPG, Pheochromocytoma and Paraganglioma; PRAD Prostate adenocarcinoma; Rectum adenocarcinoma; SARC, Sarcoma; SKCM, Skin Cutaneous Melanoma; STAD, Stomach adenocarcinoma; TGCT, Testicular Germ Cell Tumors; THCA, Thyroid carcinoma; THYM, Thymoma; UCEC, Uterine Corpus Endometrial Carcinoma; UCS, Uterine Carcinosarcoma; UVM, Uveal Melanoma. (B) SERPINE1 protein expression in different tumors provided by UALCAN using data from Clinical Proteomic Tumor Analysis Consortium (CPTAC) and the International Cancer Proteogenome Consortium (ICPC) datasets. For each tumor type, the number of normal and tumor samples is indicated in brackets. (C) Kaplan-Meier survival plots of patients with COAD based on the high or low expression levels of *SERPINE1* mRNA from The Human Protein Atlas.

**Table S1. Number of crosslinking sites for each gene in the three studied conditions.**

**Table S2. Raw and RPKM counts localized in the crosslinking regions of each gene in the three studied conditions.**

**Table S3. Position of the crosslinking sites in the *Serpine1* mRNA in control and TGF-β-treated cells.**

**Table S4. Transcriptomic profile of NMuMG control cells and NMuMG cells treated with TGF-β for 2 hours.**

**Table S5. Regions of specific crosslinking sites of *Serpine1* mRNA in TGF-β-treated cells.**

**Table S6. Number of miRNA binding sites in the mRNA *Serpine1* crosslinking regions.**

**Table S7. List of the 16 Differentially Regulated Alternative Splicing Events (from 16 Distinct Genes) (Serpine1ATG* vs RelControl).**

**Table S8. List of the 21 Differentially Regulated Alternative Splicing Events (from 21 Distinct Genes) (Tra2b vs RelControl).**

**Table S9. Transcriptomic profile of *Serpine1ATG**-overexpressing NMuMG cells versus RelControl NMuMG cells. KEGG, Reactoma and GO analysis.**

**Table S10. Transcriptomic profile of *Tra2b*-overexpressing NMuMG cells versus RelControl NMuMG cells. KEGG, Reactoma and GO analysis.**

**Table S11. Primers and Oligonucleotides Related to Experimental Procedures**

**Movie S1. Wound-healing assay.** Time-lapse video microscopy of RelControl, *Serpine1ATG**-expressing and *Serpine1wt*-expressing NMuMG cells.

**Movie S2. Wound-healing assay.** Time-lapse video microscopy of control and *Serpine1***^-/-^** NMuMG cells.

**Movie S3.** **Wound-healing assay**. Time-lapse video microscopy of *Serpine1***^-/-^** NMuMG cells and *Serpine1***^-/-^** NMuMG cells expressing *Serpine1wt* and *Serpine1ATG*.*
