# Supplementary figures and images for: Serpine1 mRNA confers mesenchymal characteristics to the cell and promotes CD8+ T cells exclusion from colon adenocarcinomas
Source: Cell Death Discov. 2024 Mar 6;10:116. doi: 10.1038/s41420-024-01886-8 (PMC10917750; doi:10.1038/s41420-024-01886-8)

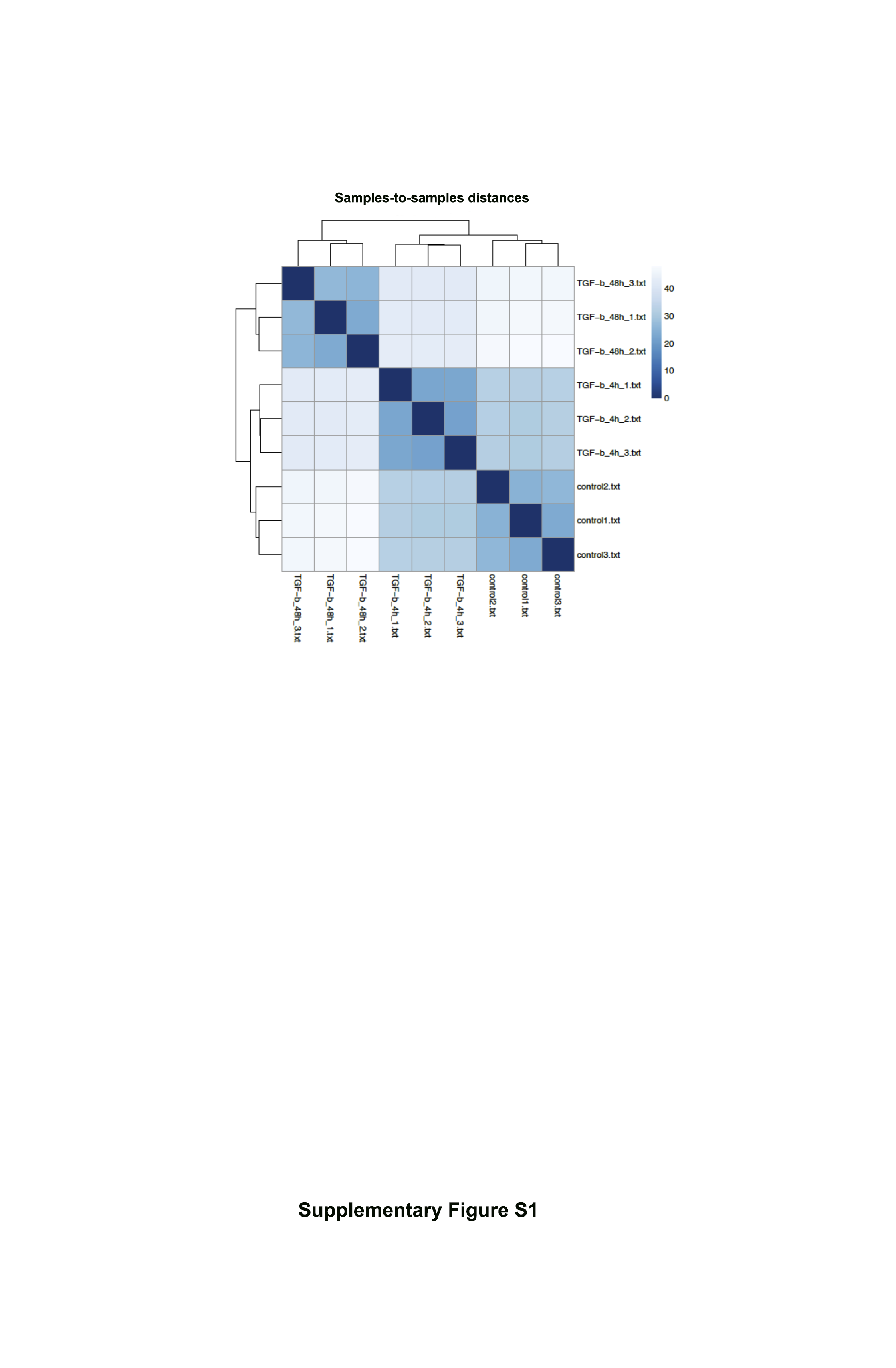

Supplement: Supplementary file 2 — Supplementary Figure S1 [file 41420_2024_1886_MOESM2_ESM.png]

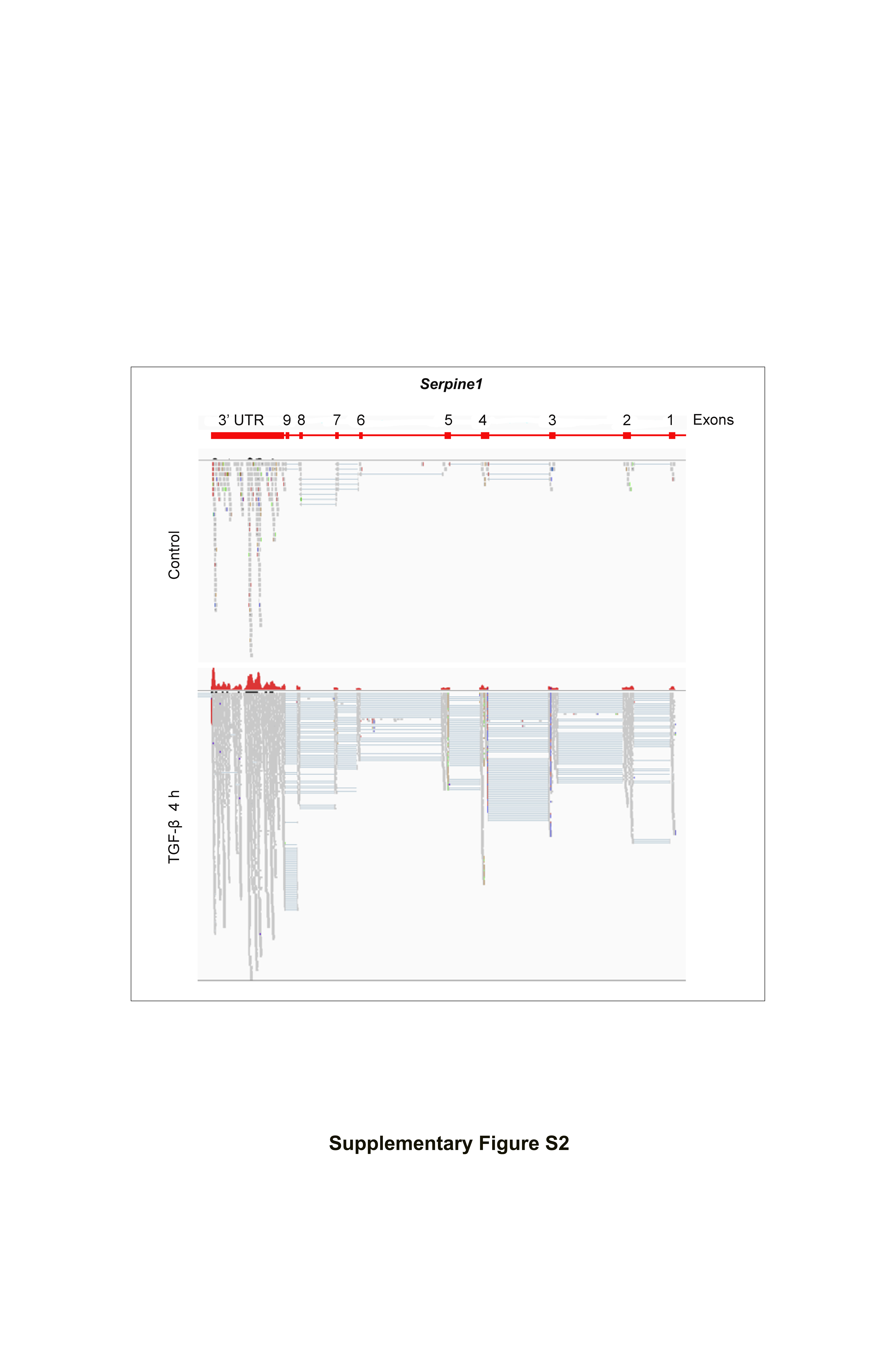

Supplement: Supplementary file 3 — Supplementary Figure S2 [file 41420_2024_1886_MOESM3_ESM.png]

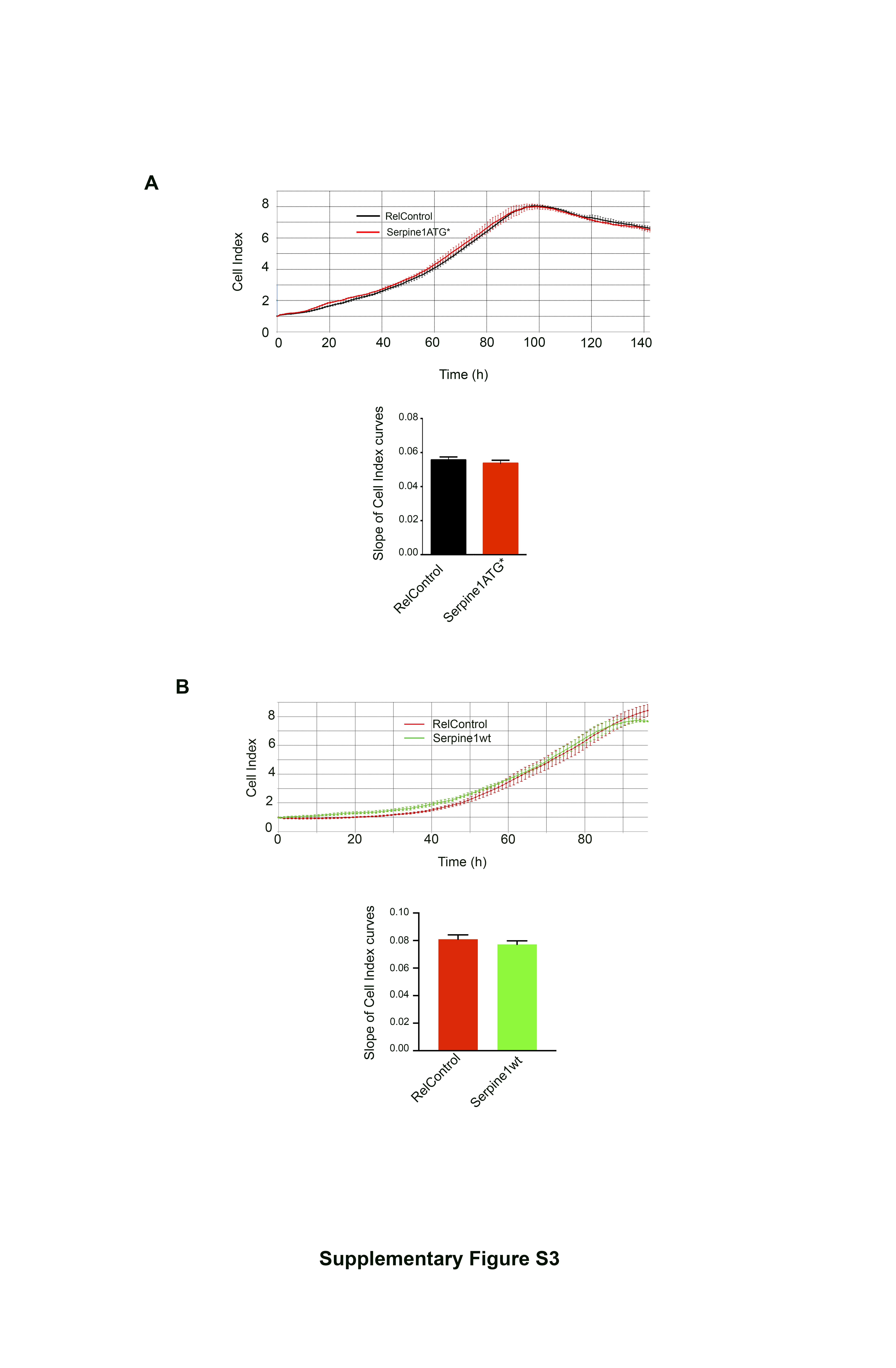

Supplement: Supplementary file 4 — Supplementary Figure S3 [file 41420_2024_1886_MOESM4_ESM.png]

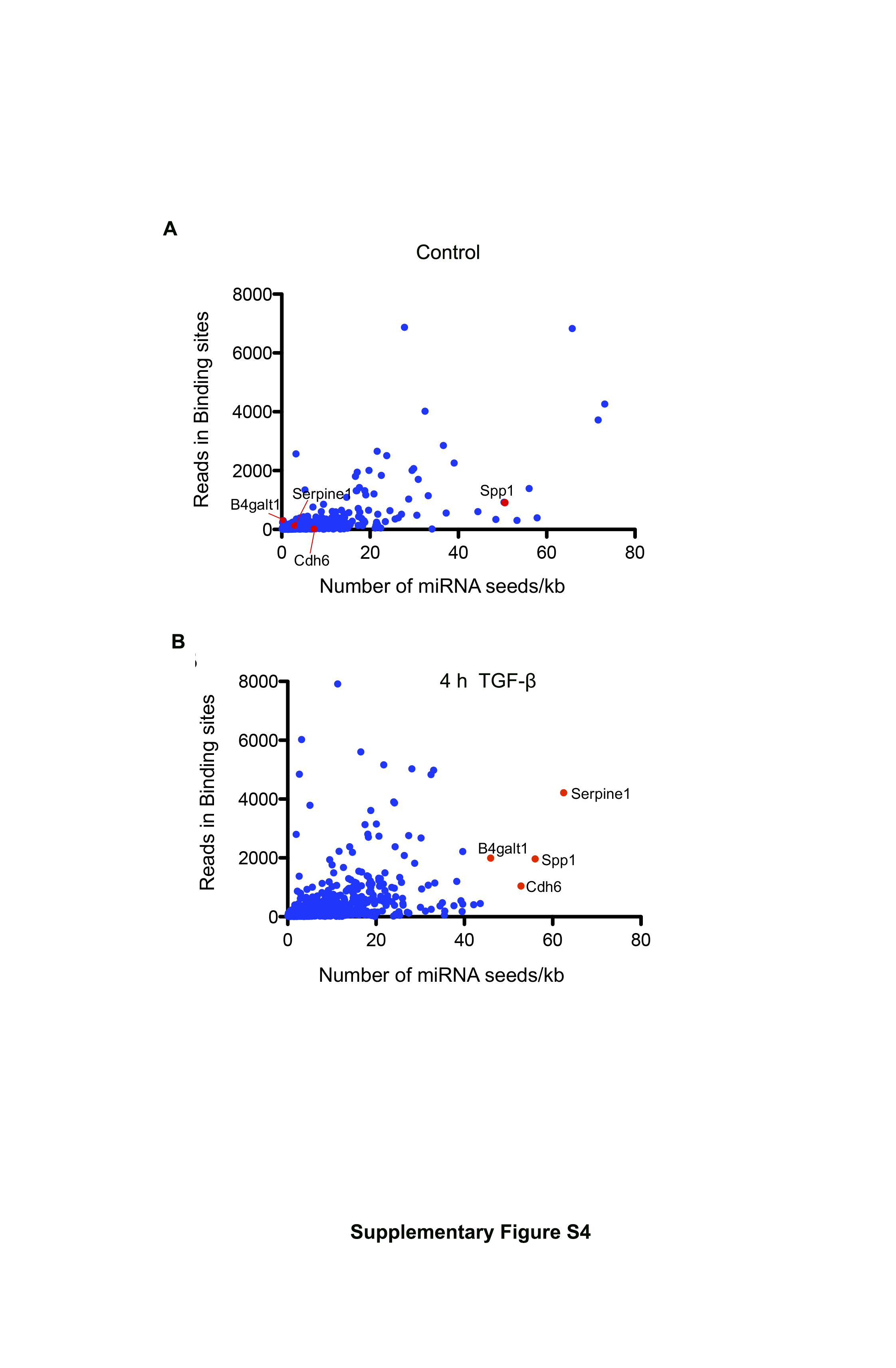

Supplement: Supplementary file 5 — Supplementary Figure S4 [file 41420_2024_1886_MOESM5_ESM.png]

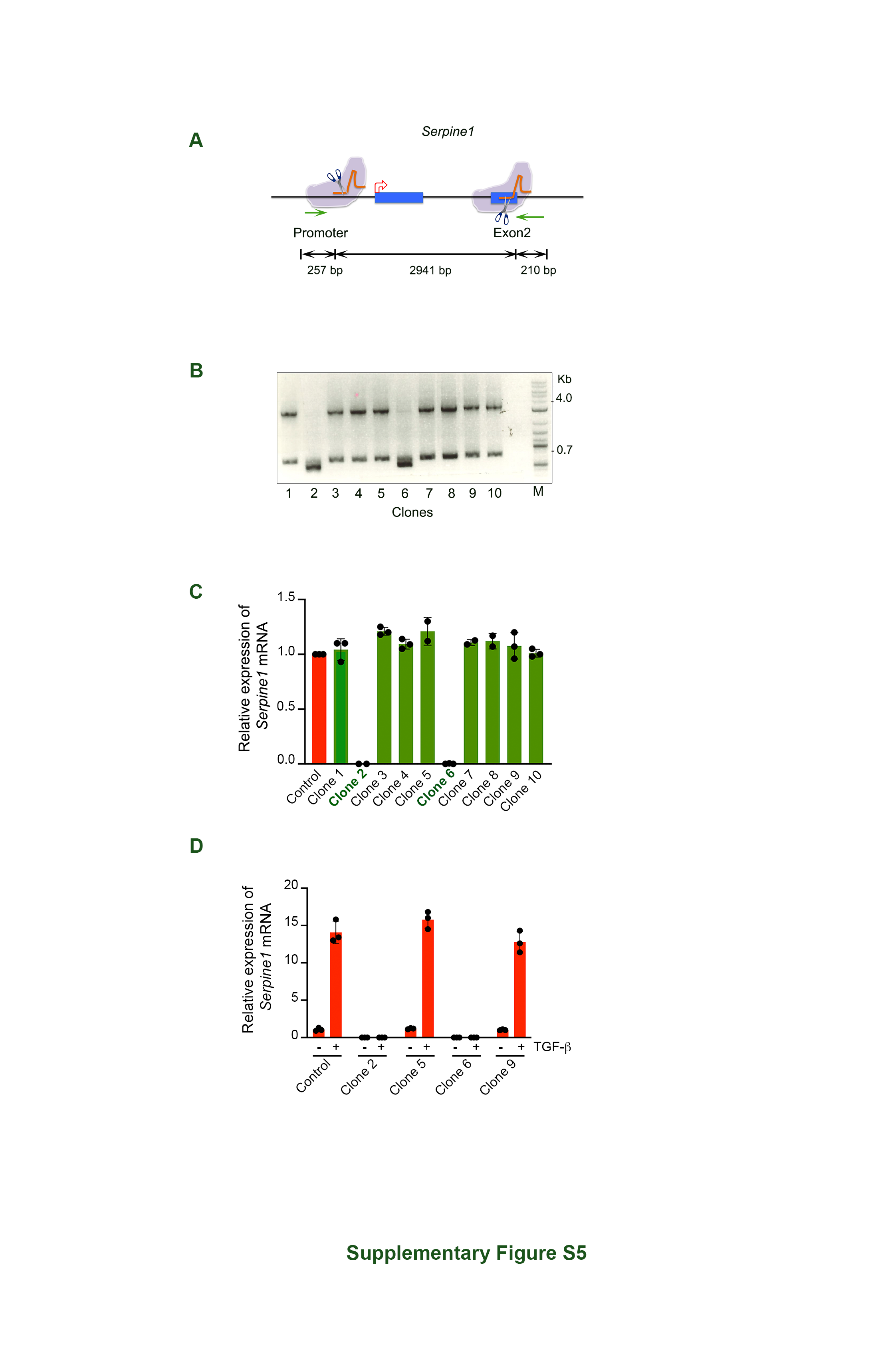

Supplement: Supplementary file 6 — Supplementary Figure S5 [file 41420_2024_1886_MOESM6_ESM.png]

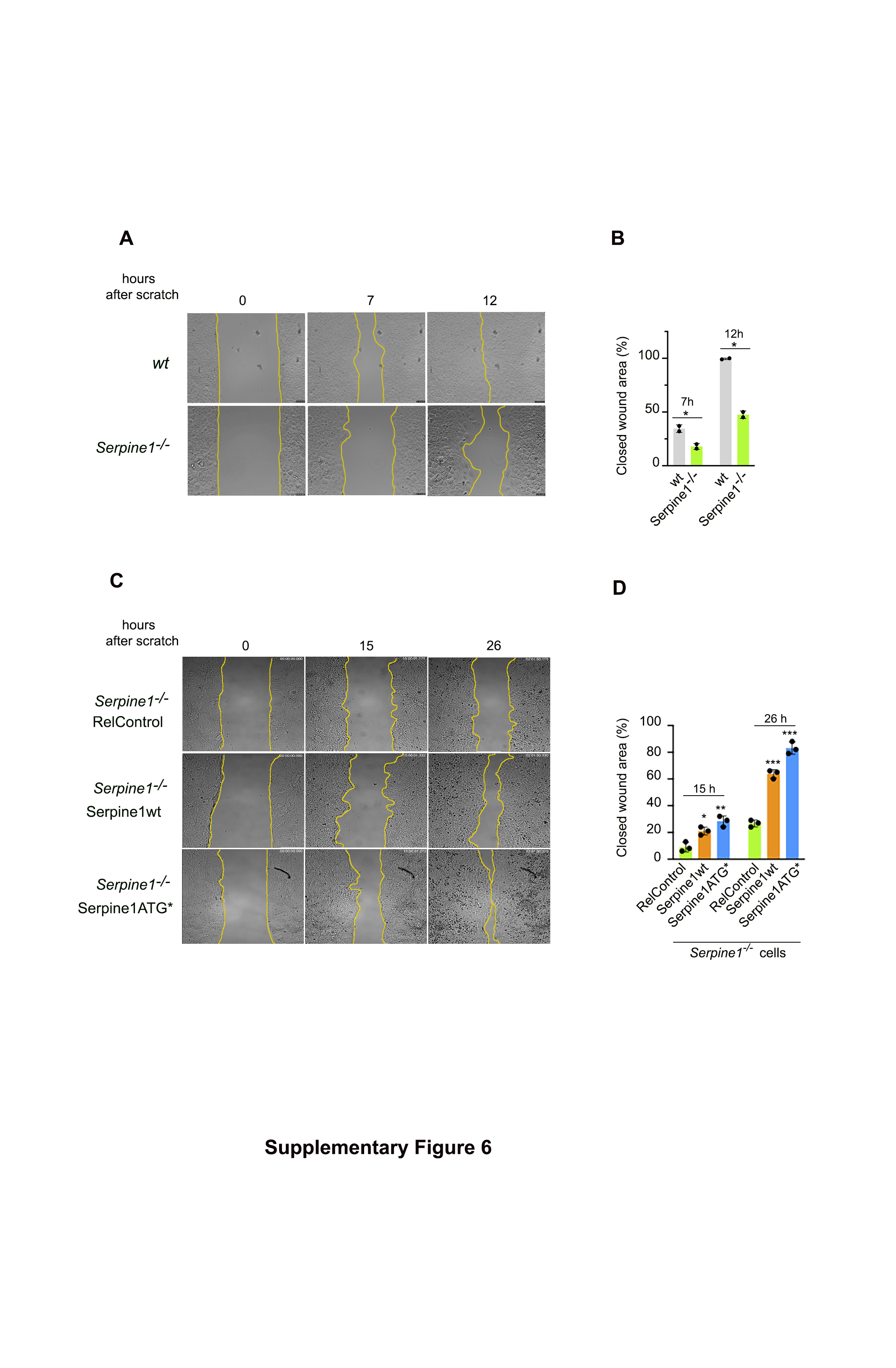

Supplement: Supplementary file 7 — Supplementary Figure S6 [file 41420_2024_1886_MOESM7_ESM.png]

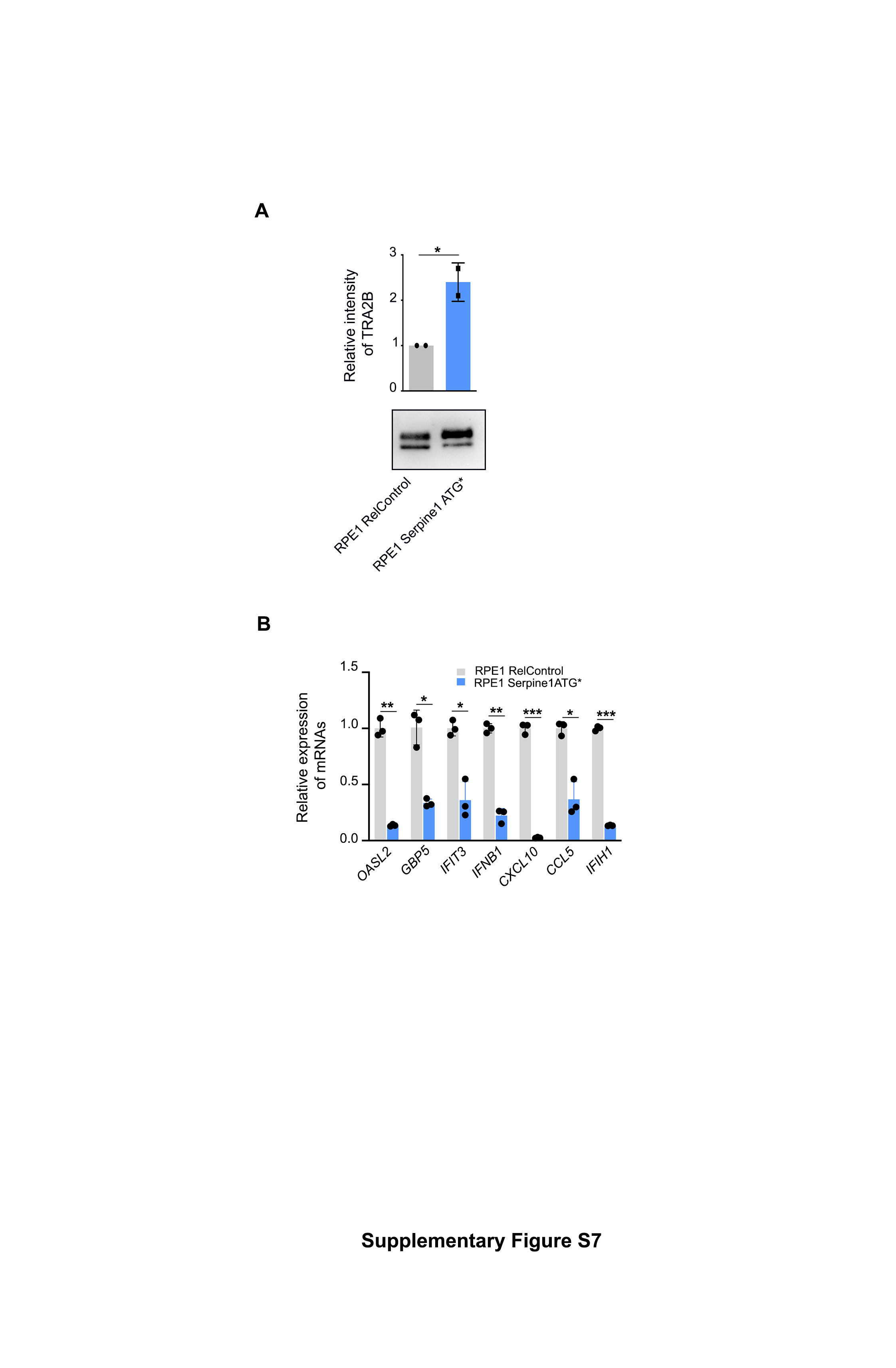

Supplement: Supplementary file 8 — Supplementary Figure S7 [file 41420_2024_1886_MOESM8_ESM.png]

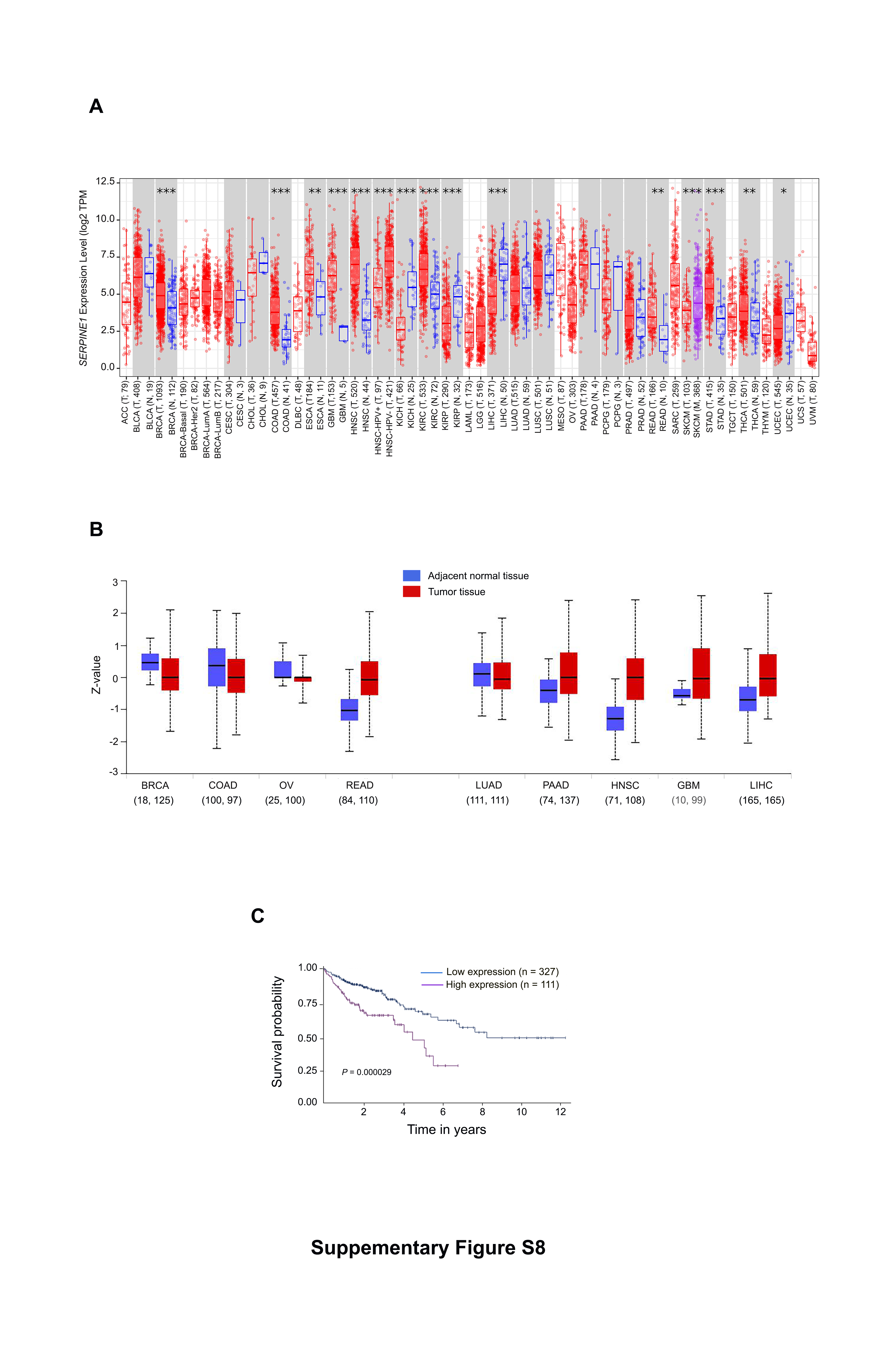

Supplement: Supplementary file 9 — Supplementary Figure S8 [file 41420_2024_1886_MOESM9_ESM.png]

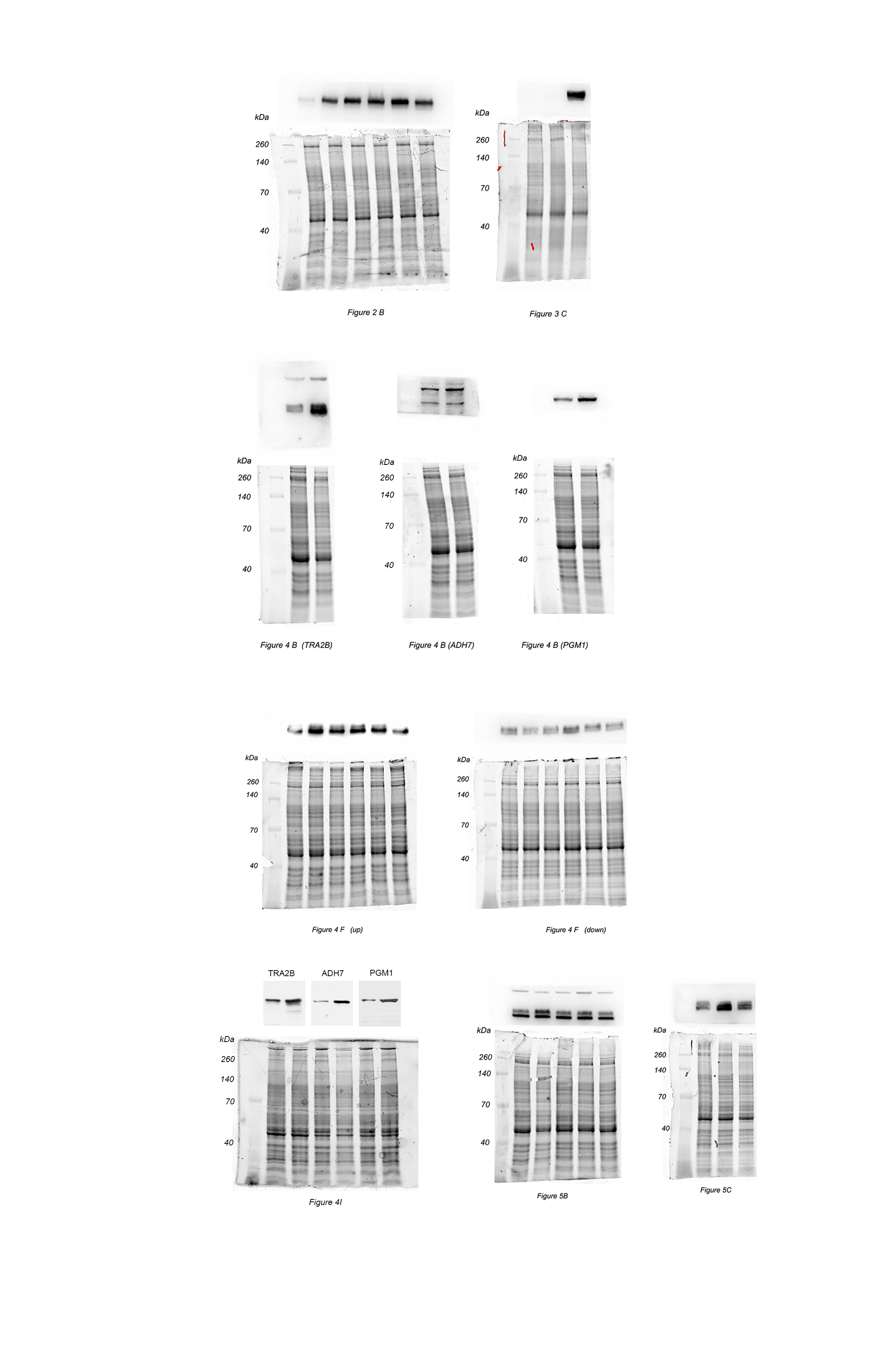

Supplement: Supplementary file 24 — Original Data File [file 41420_2024_1886_MOESM24_ESM.png]
