# Supplementary material for: Serpine1 mRNA confers mesenchymal characteristics to the cell and promotes CD8+ T cells exclusion from colon adenocarcinomas
Source: Cell Death Discov. 2024 Mar 6;10:116. doi: 10.1038/s41420-024-01886-8 (PMC10917750; doi:10.1038/s41420-024-01886-8)
Supplement: Supplementary file 25 — aj-checklist [file 41420_2024_1886_MOESM25_ESM.pdf]

Corresponding Author Name: Jose A. Pintor-Toro
Manuscript Number:_CDDISCOVERY-23-1734-T

SPRINGER NATURE

Reporting Summary

Springer Nature wishes to improve the reproducibility of the work that we publish. This checklist is used to ensure good reporting standards and to improve the reproducibility. Please respond completely to all questions relevant to your manuscript. For more information, please read the journal's Guide to Authors.

☑ **Check here to confirm that the following information is available in the Material & Methods section:**

- **The exact sample size (*n*)** for each experimental group/condition, given as a number, not a range
- **A description of the sample collection** allowing the reader to understand whether the samples represent technical or biological replicates (including how many animals, litters, culture, etc.)
- **A statement of how many times the experiment shown was replicated in the laboratory**
- **Definitions of statistical methods and measures**: For small sample sizes (n<5) descriptive statistics are not appropriate, instead plot individual data points
    - Very common tests, such as *t*-test, simple χ^2 tests, Wilcoxon and Mann-Whitney tests, can be unambiguously identified by name only, but more complex techniques should be described in the methods section
    - Are tests one-sided or two-sided?
    - Are there adjustments for multiple comparisons?
    - **Statistical test results**, e.g., ***P* values**
    - Definition of **'center values'** as **median or mean**;
    - Definition of **error bars as s.d. or s.e.m. or c.i.**

Please ensure that the answers to the following questions are reported in the manuscript itself. We encourage you to include a specific subsection in the methods section for statistics, reagents and animal models. Below, provide the page number or section and paragraph number.

Statistics and general methods

	**Reported in section/paragraph or page #**
1. How was the sample size chosen to ensure adequate power to detect a pre-specified effect size? (Give section/paragraph or page #)	Samples size for each experiment is indicated in the figure legends. The sample size was chosen based on previous experience in the lab, for each experiment to yield high [illegible]
For animal studies, include a statement about sample size estimate even if no statistical methods were used.	No animal estudies were performed in this work
2. Describe inclusion/exclusion criteria if samples or animals were excluded from the analysis. Were the criteria pre-established? (Give section/paragraph or page #)	No data were excluded from the analyses
3. If a method of randomization was used to determine how samples/animals were allocated to experimental groups and processed, describe it. (Give section/paragraph or page #)	This study does not involve randomization of samples, following standard procedures generally employed in other relevant similar studies. See for example Partridge et al., 2020 [illegible]
For animal studies, include a statement about randomization even if no randomization was used.	No animal estudies were performed in this work

4. If the investigator was blinded to the group allocation during the experiment and/or when assessing the outcome, state the extent of blinding. (Give section/paragraph or page #)	Investigators were not blinded for selection of cell culture plates used in the treatments (TGFbeta-treatments) following standard procedures generally employed in other relevant similar studies. See for example Partridge et [illegible]
For animal studies, include a statement about blinding even if no blinding was done.	No animal estudies were performed in this work
5. For every figure, are statistical tests justified as appropriate?	Statistical test used is indicated in the figure legends
Do the data meet the assumptions of the tests (e.g., normal distribution)?	Normal distribution was verified using Kolmogorov-Smirnov test.
Is there an estimate of variation within each group of data?	Standard deviations were calculated
Is the variance similar between the groups that are being statistically compared? (Give section/paragraph or page #)	No stimation of variation was performed. Different variance between groups was assumed.

Reagents

	Reported in section/paragraph or page #
6. Report the source of antibodies (vendor and catalog number)	anti-TRA2B (Santa Cruz; sc166829)<br>anti-PGM1 (Invitrogen; PA5-55008)<br>[illegible]
7. Identify the source of cell lines and report if they were recently authenticated (e.g., by STR profiling) and tested for mycoplasma contamination	NMuMG cell line was provided by Dr. A. García-Herreros (commercial source: ATCC). To ensure reproducibility, cultures were grown no longer than 2 months and monitored by PCR to prevent mycoplasma contamination. [illegible]

Animal Models

	Reported in section/paragraph or page #
8. Report species, strain, sex and age of animals	No animal estudies were performed in this work
9. For experiments involving live vertebrates, include a statement of compliance with ethical regulations and identify the committee(s) approving the experiments.	n/a

10. We recommend consulting the ARRIVE guidelines (*PLoS Biol.* **8**(6), e1000412,2010) to ensure that other relevant aspects of animal studies are adequately reported.

Human subjects

		Reported in section/paragraph or page #
11.	Identify the committee(s) approving the study protocol.	Medical Ethics Committee of Virgen del Rocío Virgen Macarena Hospitals (Sevilla)
12.	Include a statement confirming that informed consent was obtained from all subjects.	Surgical samples were obtained from patients who were previously informed and gave their consent, as established by [illegible]
13.	For publication of patient photos, include a statement confirming that consent to publish was obtained.	n/a
14.	Report the clinical trial registration number (at ClinicalTrials.gov or equivalent).	n/a

15. For phase II and III randomized controlled trials, please refer to the CONSORT statement and submit the CONSORT checklist with your submission.

16. For tumor marker prognostic studies, we recommend that you follow the REMARK reporting guidelines.

Data deposition

		Reported in section/paragraph or page #
17.	Provide accession codes for deposited data. Data deposition in a public repository is mandatory for:<br>a. Protein, DNA and RNA sequences<br>b. Macromolecular structures<br>c. Crystallographic data for small molecules<br>d. Microarray data	NGS data are available at the NCBI Gene Expression Omnibus (GEO) under the accession number GSE221153 without restrictions. GSE221152 and GSE221076 correspond to accession numbers of iCLIP data and RNA-seq data respectively.

Deposition is strongly recommended for many other datasets for which structured public repositories exist; more details on our data policy are available in the Guide to Authors. We encourage the provision of other source data in supplementary information or in unstructured repositories such as Figshare and Dryad. We encourage publication of Data Descriptors (see Scientific Data) to maximize data reuse.

18.	If computer code was used to generate results that are central to the paper's conclusions, include a statement in the Methods section under "**Code availability**" to indicate whether and how the code can be accessed. Include version information as necessary and any restrictions on availability.	n/a
